# Mapping and identification of *CsUp,* a gene encoding an Auxilin-like protein, as a putative candidate gene for the *upward-pedicel* mutation (*up*) in cucumber

**DOI:** 10.1186/s12870-019-1772-4

**Published:** 2019-04-25

**Authors:** Jingxian Sun, Tingting Xiao, Jingtao Nie, Yue Chen, Duo Lv, Ming Pan, Qifan Gao, Chunli Guo, Leyu Zhang, Huan-Le He, Hongli Lian, Junsong Pan, Run Cai, Gang Wang

**Affiliations:** School of Agriculture and Biology, Shanghai Jiao Tong University, 800 Dongchuan Road, Minhang District, Shanghai, 201100 China

**Keywords:** *Cucumis sativus*, Upward-pedicel, Map-based cloning, Pedicel orientation, Auxilin-like protein

## Abstract

**Background:**

Pedicel orientation can affect the female flower orientation and seed yield in cucumber.

A spontaneous mutant possessing upward growth of pedicels was identified in the wild type inbred strain 9930 and named *upward-pedicel* (*up*). The morphological and genetic analyses of *up* were performed in this study. In order to clone the *up* gene, 933 F_2_ individuals and 524 BC_1_ individuals derived from C-8-6 (WT) and *up* were used for map-based cloning.

**Results:**

*up* was mapped to a 35.2 kb physical interval on chromosome 1, which contains three predicted genes. Sequencing analysis revealed that a 5-bp deletion was found in the second exon of *Csa1G535800,* and it led to a frameshift mutation resulting in a premature stop codon. The candidate gene of *CsUp* (*Csa1G535800*) was further confirmed via genomic and cDNA sequencing in biparental and natural cucumber populations. Sequencing data showed that a 4-bp deletion was found in the sixth exon of *Csa1G535800* in CGN19839, another inbred line, and there was also a mutation of an amino acid in *Csa1G535800* that could contribute to the upward growth of pedicels in CGN19839. Moreover, it was found that *Csa1G535800* exhibited strong expression in the pedicel of WT, suggesting its important role in development of pedicel orientation. Thus, *Csa1G535800* was considered to be the candidate gene of *CsUp*.

**Conclusions:**

*CsUp* encodes an Auxilin-like protein and controls pedicel orientation in cucumber. The identification of *CsUp* may help us to understand the mechanism of pedicel orientation development and allow for investigation of novel functions of Auxilin-like proteins in cucumber.

**Electronic supplementary material:**

The online version of this article (10.1186/s12870-019-1772-4) contains supplementary material, which is available to authorized users.

## Background

Cucumber (*Cucumis sativus* L., 2n = 2x = 14) is an economically important vegetable crop cultivated worldwide. It was first domesticated in India and is currently widely grown in China [1–4]. In cucumber, most organic nutrients of stalk and fruit was transported in via the pedicel [5]. The orientation of the pedicel, a critical organ in various plants, is also an important plant architectural trait that can influence flower position and orientation and thus affect pollination, pollinator attraction, insect foraging behaviour, pollen transfer and seed production [6–14].

In recent years, pedicel orientation has been studied extensively. In *Arabidopsis*, *CRM1*/*BIG*, an auxin transport-related gene, was found to encode a membrane-associated protein affecting pedicel orientation [15–20]. In the *crm1/big* mutant, the pedicels and internodes were very short and downward-bending [15, 19]. In addition to *CRM1*/*BIG*, several genes in *Arabidopsis* were also reported to be involved in regulating pedicel orientation, including *BP*, *KNAT2*, *KNAT6*, *AS1*, *AS2*, *ATH1 and LEAFY* [16, 17, 20–27]. In tobacco, *NtBPL*, the homologous gene of *BP* in *Arabidopsis*, was found to be involved in pedicel elongation but its effect on pedicel orientation is limited [16, 28]. In tomato, overexpression of *SlAGO7* could lead to a dramatic change in pedicel morphology and cause the pedicels to be upward-pointing [29]. In pepper, *Capana12g000943* was predicted to be an important player in pedicel orientation development by transporting cytokinin across the plasma membrane [30]. However, in cucumber, most studies were mainly focused on flower sex determination, fruit skin colour, compact plant architecture, powdery mildew resistance, and trichomes formation. The mechanism of pedicel orientation development remains elusive.

Most cucumber inbred lines possess downward growth of pedicels with downward-facing or horizontal-facing female flowers. In the present study, a spontaneous upward-pedicel mutant named *upward-pedicel (up)* was derived from wild type 9930. The pedicel of *up* always displays upward growth with an upward-facing flower until 5 days after pollination (DAP). After 5 DAP, the pedicel turns downward in response to the increasing fruit weight. In this study, we report the map-based cloning of *up* and demonstrate that it encodes an Auxilin-like protein that might play an important role in pedicel orientation development in cucumber. This finding can help us to understand the mechanism of pedicel orientation development in cucumber.

## Results

### The *up* phenotype was controlled by a recessive nuclear gene in cucumber

**Fig. 1:**
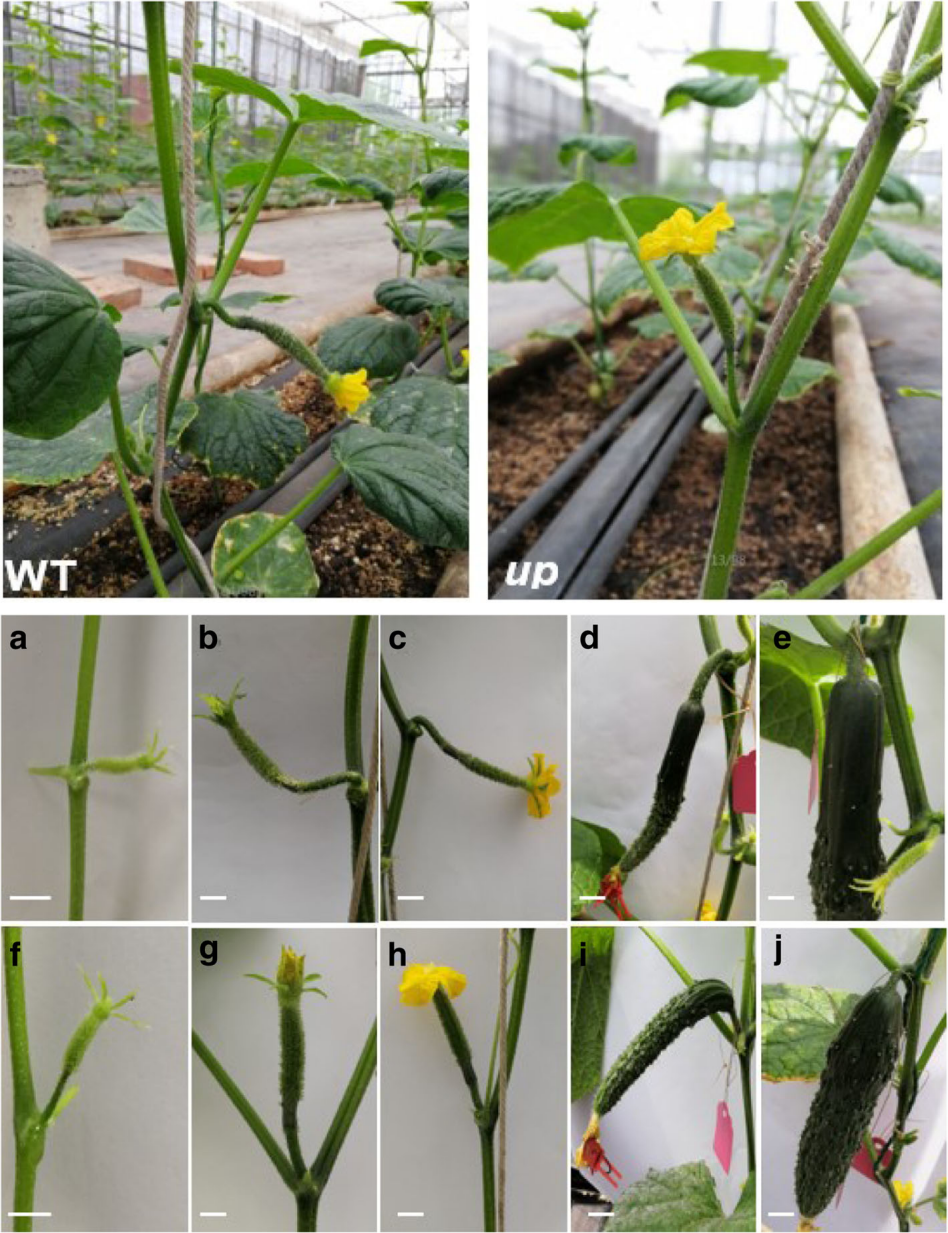
Phenotypic features of the pedicel in WT and *up*, and the pedicel orientation at different stages in two parents. **a**, **b**, **c**, **d** and **e** are pedicels from WT. **f**, **g**, **h**, **i** and **j** are pedicels from *up*. **a**, **f** Young fruits at an early stage (approximately 1 cm); **b**, **g** Young fruits of the day before female flowers open; **c**, **h** Young fruits with opening female flowers; **d**, **i** Fruits 3 days after pollination; **e**, **j** Fruits 5 days after pollination. When the female flowers were blooming or bloomed, the pedicel was downward growth in WT and upward growth in *up*

In WT, the pedicel orientation remained horizontal when the young fruits were at the early stage. After that, their pedicel became downward-bending and the orientation of the pedicel remained downward-pointing (Fig. 1a, b, c, d and e). In *up*, the pedicel showed upward growth before 3 days after pollination and then the pedicel became downward-bending with the increasing fruit weight (Fig. 1f, g, h, i and j).

The pedicel orientation of the F_1_ plants produced from the cross of *up* and WT was the same as WT. No significant phenotypic difference was found between homozygous dominant (*Up / Up*) and heterozygous (*Up/up*) plants. In a small F_2_ population with 133 individuals, the downward-pedicel plants and upward-pedicel plants fitted a segregation ratio of 3:1 (100:33). In a large BC_1_ population with 524 individuals, the downward-pedicel plants and upward-pedicel plants fitted a segregation ratio of 1:1 (255:269). In a large F_2_ population with 800 individuals, there were 599 downward-pedicel individuals and 201 upward-pedicel individuals, which also fitted the 3:1 segregation ratio (Table.1). These results suggested that both the F_2_ population and the BC_1_ population conformed to Mendel segregation which confirmed that *up* was conferred by a single recessive locus in cucumber.

**Table 1:** Segregation analysis of the *up* trait in the F_2_ population (WT × *up*)

Population	Number of plants			Expected ratio	*χ* ^2a^	*P*
	Total	D type	U type			
F_1_	20	20	0	━	━	━
F_2_	133	100	33	3:1	0.003	0.96
F_2_	800	607	193	3:1	0.003	0.33
BC_1_	524	255	269	1:1	0.374	0.54

*D*: downward-pedicel; *U*: upward-pedicel

a *χ2* (0.05, 1) = 3.84

### Primary mapping

**Fig. 2:**
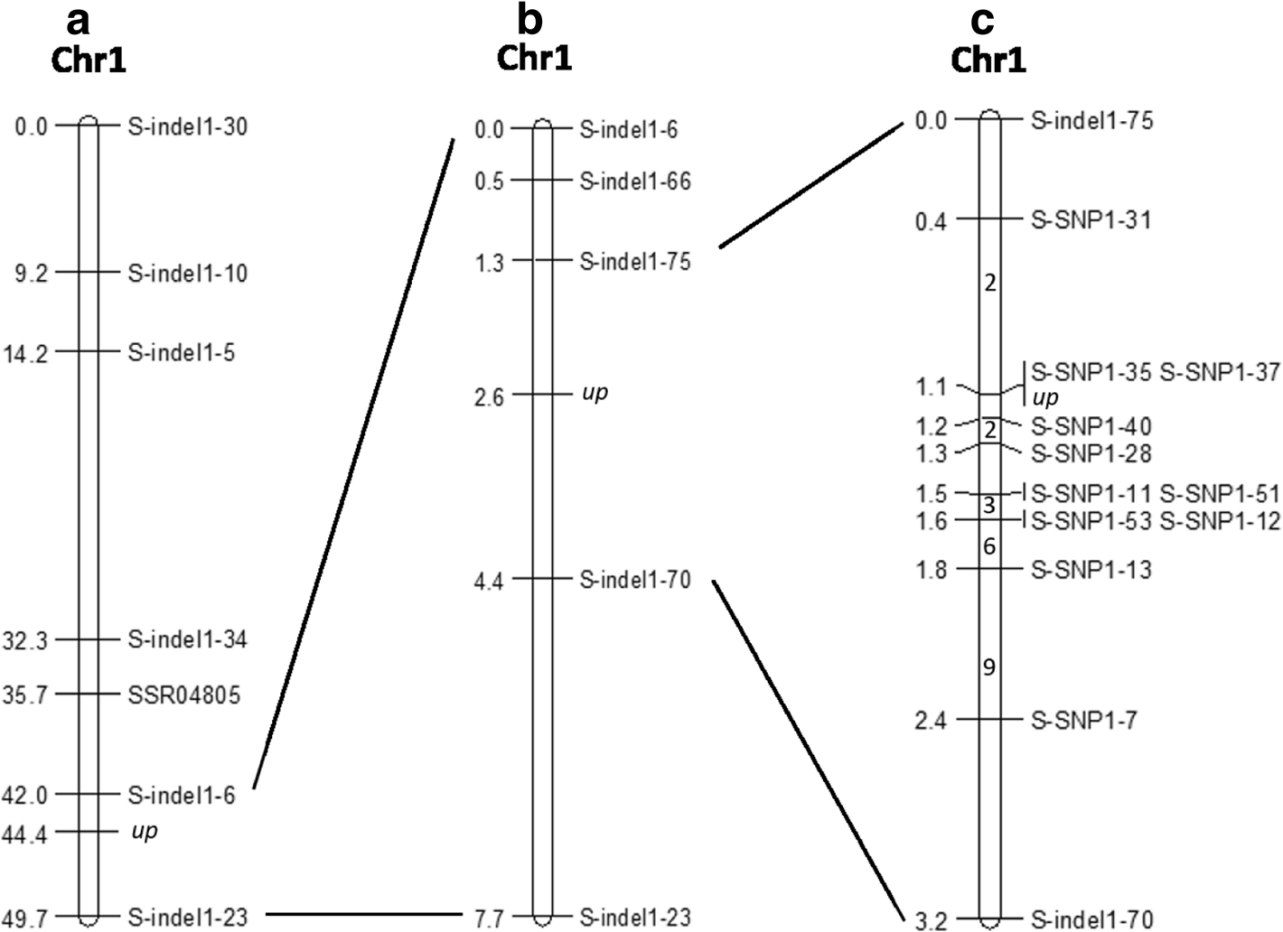
Genetic and physical maps of *up* locus. **a** *up* locus was located on chromosome 1 by using downward-pedicel individuals and upward-pedicel individuals randomly selected from the small F_2_ population (133 individuals), and *up* locus was firstly located between S-indel1–6 and S-indel1–23 using 70 F_2_ individuals which were from the small F_2_ population. **b** *up* locus was further mapped to the region between S-indel1–75 and S-indel1–70 by using the small F_2_ population (133 individuals). **c** *up* locus was finally narrowed to the region with 35.2 kb physical distance between S-SNP1–35 and S-SNP1–40, using 933 F_2_ individuals (from the small F_2_ population with 133 individuals and the large F_2_ population with 800 individuals) and 524 BC_1_ individuals for high-density genetic mapping. The number on the chromosome represent the quantity of recombinants for the corresponding marker

Based on the re-sequencing genomic data, 95 polymorphic InDel markers between *up* and WT were developed. All of the 41 polymorphic SSR markers and 95 polymorphic InDel markers between *up* and WT were screened to analyse the WT and M DNA pools using a BSA strategy. Among these markers, seven markers on cucumber chromosome 1 showed polymorphism between the WT and M DNA pool (Additional file 1: Figure S1). The seven markers were used for linkage analysis by using 70 F_2_ individuals which were randomly selected from the small F_2_ population. The results showed that the *up* locus was located on chromosome 1, flanked by the closer markers s-indel1–6 and s-indel1–23, at a genetic distance of 2.4 and 5.3 cM, respectively (Fig. 2a). Based on the genomic sequence, three new polymorphic InDel markers (S-indel1–66, S-indel1–75 and S-indel1–70) between s-indel1–6 and s-indel1–23 were developed and the three markers were used for further mapping. The *up* locus was flanked by closer markers s-indel1–75 and s-indel1–70. These two markers (s-indel1–75 and s-indel1–70) were used to screen the F_2_ population (small F_2_ population and large F_2_ population) and the large BC_1_ population. Finally, two markers s-indel1–75 and s-indel1–70 showed 13 and 26 recombinants among 933 F_2_ individuals and 524 BC_1_ individuals, respectively (Fig. 2b).

### Fine mapping of the *up* locus

The physical distance between s-indel1–75 and s-indel1–70 was approximately 785.1 kb according the ‘9930’ genome database (http://cucurbitgenomics.org/organism/2). The resequencing genome data showed that there were no more polymorphic InDel markers between s-indel1–75 and s-indel1–70. Therefore, SNP markers between s-indel1–75 and s-indel1–70 were developed for fine mapping. Eleven SNP markers were developed and these SNP markers showed polymorphisms in the parents. Out of 933 F_2_ individuals and 524 BC_1_ individuals, 39 recombinants between s-indel1–75 and s-indel1–70 were used for fine mapping with the polymorphic SNP markers. Finally, the *up* locus was narrowed to a region between S-SNP1–35 and S-SNP1–40 markers, encompassing a physical distance of 35.2 kb (Fig. 2c) and each of two markers with one and two recombinants, respectively. The marker S-SNP1–37 was co-segregated with the *up* locus between S-SNP1–35 and S-SNP1–40.

### Predicted genes in the *up* region

**Fig. 3:**
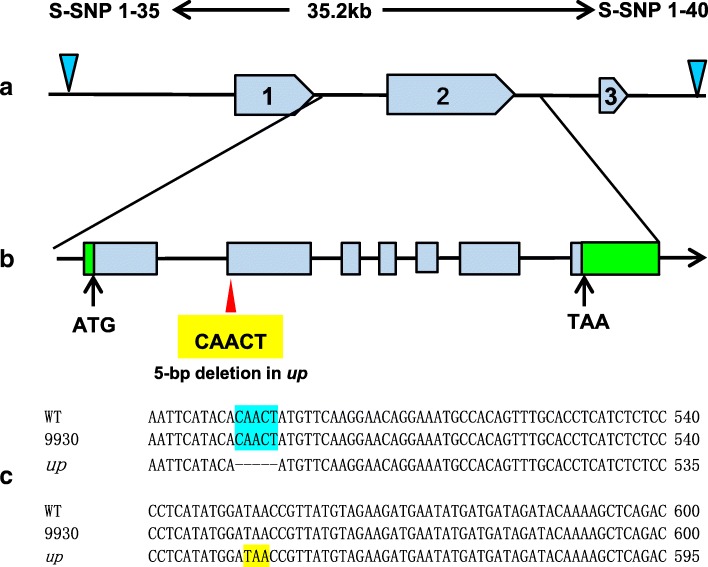
Representation of the three putative genes between S-indel1–75 and S-indel1–70. **a** There were three putative genes (*blue boxes*) between S-SNP1–35 and S-SNP1–40. Gene numbers correspond to those in Table 2. **b** Structure of *Csa1G535800*. Boxes and lines indicate exons and introns, respectively. Two green boxes in *Csa1G535800* indicate the 5′ UTR and the 3′ UTR. Sequencing results revealed that, compare to WT, *up* has a 5-bp deletion at 882–886 bp from the ATG start codon in the second exon of *Csa1G535800*. The yellow box shows the deletion sequence in *up*. **c** The position of the 5-bp deletion in the coding sequence. Sequencing results revealed that there was a 5-bp deletion at 491–495 bp from the ATG start codon that resulted in a premature stop codon at 612–614 bp in the mRNA

**Fig. 4:**
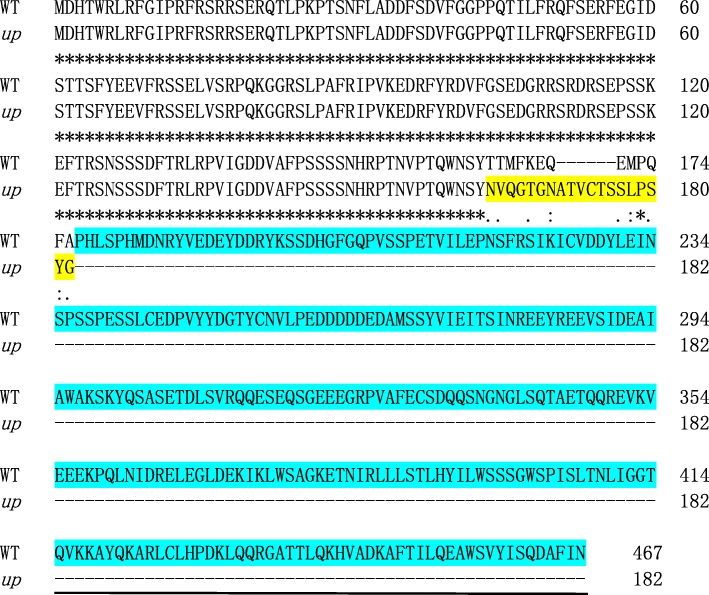
Alignment of predicted protein sequences of *Csa1G535800* between WT and *up*. The 5-bp deletion site resulted in the introduction of a premature stop codon, and the encoded protein lacked the normal amino acids from position 164. The amino acids highlighted in yellow are abnormal in *up* and the amino acids highlighted in blue are missing in *up*. The DnaJ domain at the C-terminal is underlined

In the 35.2 kb DNA segment, three putative genes were identified in the ‘9930’ genome database (http://cucurbitgenomics.org/organism/2). The detailed information about these genes is summarized in Fig. 3a and Table 2. DNA sequence of the three genes and their promoters (2000 bp before start codon) was amplified and analysed. Compared to WT, there was no polymorphism in both promoter sequence and gene sequence of *Csa1G535810* (Additional file 10: Figure S9, Additional file 13: Figure S12). In *Csa1G535790*, there was only one SNP polymorphism in the promoter region at -611 bp position (Additional file 12: Figure S11) and no polymorphism in the gene sequence (Additional file 9: Figure S8). In *Csa1G535800*, there was a 5-bp deletion in the second exon (Additional file 7: Figure S6) and no polymorphism in the promoter sequence (Additional file 11: Figure S10). cDNA sequence amplification and analysis revealed an identical 5-bp deletion at the same locus in *up* (Additional file 8: Figure S7). Further analysis revealed that the 5-bp deletion caused a frameshift mutation and resulted in a premature stop codon at 612–614 bp in the mRNA (Fig. 3c). Protein BLAST searches indicated that the gene *Csa1G535800* was predicted to encode an Auxilin-like protein with a conserved DnaJ-domain at the C-terminal (Fig. 4). Due to the 5-bp deletion, a large segment of amino acid sequence including the DnaJ domain was lost in the predicted protein (Fig. 4). Although there is a SNP in the promoter of *Csa1G535790,* we think that *Csa1G535800* was the most likely candidate gene*.*

**Table 2:** Predicted genes between markers S-SNP1–35 and S-SNP1–40

Gene No.	Predicted genes (ID)		Gene function	BLASTX plant proteins	*E*-value
	Cmb	Phytozome v10			
1	*Csa1G535790*	*Cucsa.152130*	Putative mitochondrial transcription termination factor family protein	AT2G34620	1e-128
2	*Csa1G535800*	*Cucsa.152140.1*	Auxilin-like protein	AT1G30280	4e-10
3	*Csa1G535810*	Not found	Unnamed protein	Not found	–

*ID* identification number given by cmb (http://cucurbitgenomics.org/organism/2 ) and Phytozome v10 (https://phytozome.jgi.doe.gov/pz/portal.html#!info?alias=Org_Csativus)

### Identification of the putative candidate gene for *up*

To further confirm *Csa1G535800* was the candidate gene, we examined linkage relationship between the 5-bp deletion and the *up* locus. An InDel marker (Indel-*CsUp*) developed from the fragment with the 5-bp deletion was used for genotyping the F_2_ population with 800 individuals. Genotype analysis showed that the marker Indel-*CsUp* co-segregated with the *up* locus in this F_2_ population (Additional file 2: Figure S2). Furthermore, we analysed the coding sequences of 19 cucumber inbred lines based on their resequencing data (Additional file 8: Figure S7). Among the 19 cucumber inbred lines, there was a pair of near-isogenic lines B1 (upward-pedicel) and B2 (downward-pedicel), and all of the inbred lines had downward-pedicels except B1 and CGN19839 (Additional file 3: Figure S3). Sequence analysis showed that all of the inbred lines with downward growth pedicels shared the same sequence with WT in the encoding region. Moreover, in B1, we found the same 5-bp deletion in the encoding region. In CGN19839, we found an A/G transition in exon 1 that caused an amino acid change from Ile (I) to Val (V) and a 4-bp deletion in exon 6 that led to a premature stop codon at 1243–1245 bp in the mRNA. The 4-bp deletion could also result in the DnaJ domain being lost from the predicted protein. To confirm the result, genomic DNA and cDNA of *Csa1G535800* from B1 and CGN19839 were sequenced and the results were consistent with the resequencing data.

### Phylogenetic relationships of CsUp with its homologs in other species

**Fig. 5:**
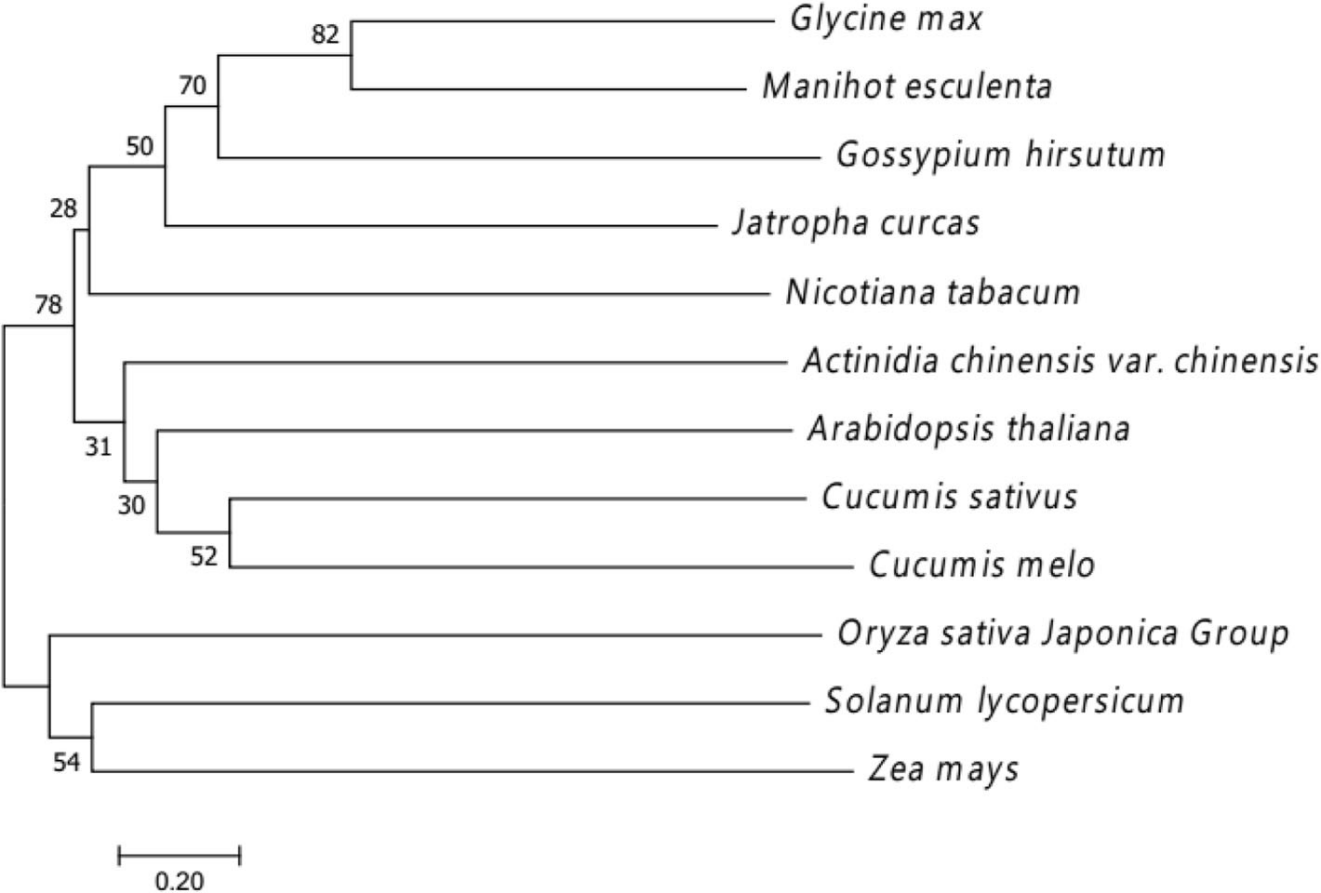
Phylogenetic tree of CsUP in cucumber and its homologs in other species. The phylogenetic tree was constructed using the neighbour-joining method built in MEGA 7, and the percentage of replicate trees in which the associated taxa clustered together in the bootstrap test (1000 replicates) are shown next to the branches. Numbers on the tree represented bootstrap values

Protein BLAST searches indicated that the gene *Csa1G535800* encodes an Auxilin-like protein (CsUp) with a conserved DnaJ-domain, and this DnaJ-domain contains 44 amino acid residues (Fig. 4). To better understand the genetic and functional relationships of CsUp between cucumber and other species, a phylogenetic tree was constructed on the basis of full-length protein sequences of CsUp and 11 other homologous proteins (Fig. 5). Amino acid sequence identity between CsUp and other homologs varied from 40 to 95%. In the phylogenetic tree, the CsUp was more similar to *LOC103487941* (*Cucumis melo*) and *At1G30280* (*Arabidopsis thaliana*). In *Cucumis melo*, *LOC103487941* is an uncharacterized protein and its function is unclear. In *Arabidopsis*, *At1G30280* is a chaperone DnaJ-domain superfamily protein named Auxilin-like7, however, limited work has been performed on the functional characterization of this gene.

### Transcript expression analysis of *Csa1G535800*

**Fig. 6:**
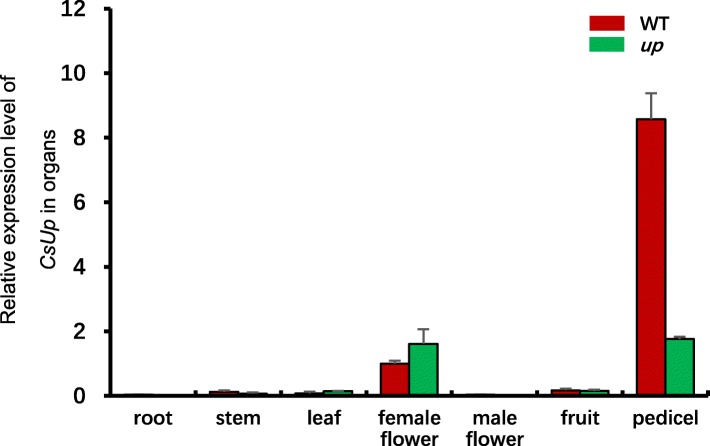
Expression level of *CsUp* candidate gene in seven organs of WT and *up.* Analysis of relative transcript abundance used female flowers in WT as the reference. Data are displayed as the ratio of expression to CsActin3 with three biological replicates. *Error* bars represent standard error (SE)

**Fig. 7:**
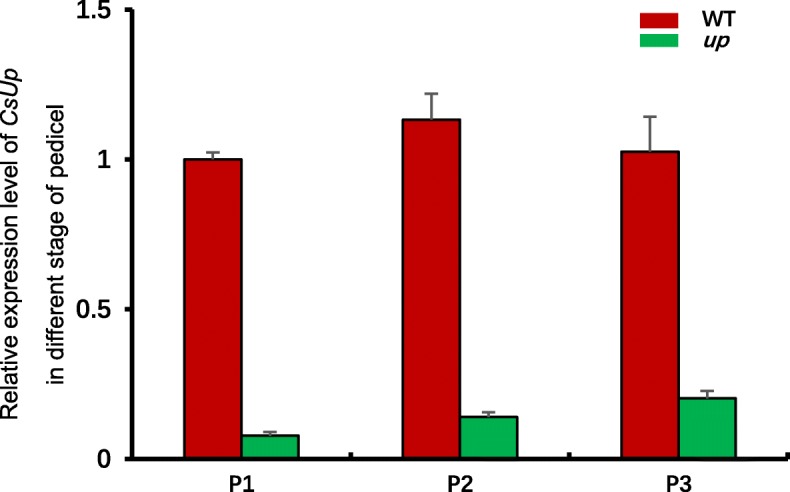
Expression level of *CsUp* candidate gene in different stage of pedicel. P1 pedicels from 1 to 1.5 cm length young fruits; P2 pedicels from the young fruits the day before the female flowers open; P3 pedicels from the young fruits with opening female flowers. Data are displayed as the ratio of expression to CsActin3 with three biological replicates. *Error* bars represent standard error (SE)

Pattern expression of *Csa1G535800* was examined in roots, stems, true leaves, female flowers, male flowers, fruits and pedicels from WT and *up* by qRT-PCR (Fig. 6). *Csa1G535800* was mainly expressed in the pedicel and female flower. The expression level showed no significant differences between WT and *up* in roots, stems, true leaves, female flowers, male flowers or fruits. In *up*, the expression level in the pedicel was significantly lower than that in WT. The expression level in WT was approximately 6.5-fold higher than that in *up*. We examined the temporal expression of *Csa1G535800* in the pedicels that were derived from 1 to 1.5 cm length fruits, the young fruits of the day before female flowers open and the young fruits with opening female flowers (Fig. 7). The expression results showed that, within WT and *up*, there was no significant difference temporally among the three stages. However, compared to WT, the expression level was significantly lower in *up* at the three stages, which is consistent with the results of pattern expression.

The temporal expression of *Csa1G535790* and *Csa1G535810* was also examined in the pedicels that were derived from 1 to 1.5 cm length fruits, the young fruits of the day before the female flowers open and the young fruits with opening female flowers (Additional file 14: Figure S13). Within WT and *up*, the transcription level of *Csa1G535790* and *Csa1G535810* was no clear difference among the three stages, and there was also no significant difference between WT and *up*. Therefore, the SNP polymorphism from the promoter region of *Csa1G535790* may not influence the gene expression level.

## Discussion

In this study, we isolated the candidate gene (*CsUp*) for upward-pedicels based on map cloning using a spontaneous upward-pedicel mutant ‘*up*’. Sequencing results revealed that a 5-bp deletion was found in the second exon of *CsUp,* and this deletion resulted in loss of a large segment of amino acids in the predicted protein (Fig. 3). After analysing the F_2_ population with 800 individuals, we found that the marker indel-*CsUp* was co-segregated with the upward-pedicel phenotype (Additional file 2: Figure S2). In B1 (upward-pedicel), the same 5-bp deletion was found in *CsUp,* but this deletion did not exist in its near-isogenic line B2 (downward-pedicel). Furthermore, a different mutation type was found in another *up* inbred line CGN19839. Sequencing data showed that one A/G transition at exon 1 caused an amino acid change from Ile (I) to Val (V) and a 4-bp deletion in the exon 6 of *CsUp* led to a premature stop codon (Additional file 4: Figure S4). We speculated that the two kinds of mutations can cause dysfunction of *CsUp* and result in the upward-pedicel phenotype in *up,* B1 and CGN19839. In WT, the expression level of *CsUp* was the highest in the pedicel, which supported *CsUp* being the most likely candidate gene of *up* (Figs. 6 and 7).

Although there was a SNP polymorphism in the promoter region of *Csa1G535790* (Additional file 12: Figure S11), while there was no clear transcription difference between WT and *up* among the three stages (Additional file 14: Figure S13). Therefore, we think the SNP polymorphism in the promoter region of *Csa1G535790* may not the reason of *up* mutant phenotype.

Pedicel is an important plant architecture trait and its orientation affects flower orientation. In other plants, flower orientation is an important characteristic that influences reproductive success and yield. In the plants with complex inflorescences, the flower orientation affects pollinator behaviour and pollination efficiency [14]. In single flower plants, the flower orientation often plays an important role in protecting flowers from bad environments [8, 9]. As single flower plants, most cucumber inbred lines present their female flowers horizontally or downward-facing. Under natural growth conditions, female flowers may employ the same protective strategy as found in other single flower plants. In *up*, when the upward-facing female flowers are blooming in natural growth conditions, the pistil stigma will lose the protection of the petals and may be vulnerable to rain drops or direct sunlight. The reproduction of cucumbers may be influenced by bad weather. We speculated that the horizontal or downward-facing female flowers in cucumber are probably due to natural selection.

To date, a number of underlying molecular regulators controlling the pedicel orientation have been uncovered, including *Arabidopsis LEAFY* [27], *KNAT1/BP* [16, 21], *KNAT2* and *KNAT6* [20], *CRM1/BIG* [19], *AS1* [17], *AS2* [31] and *ATH1* [23]; tomato *SlAGO7* [29]; Gloxinia *TCP* [32]; and tobacco *NtSVP* [28]. However, none of their homologous proteins was identified in cucumber. In this study, we isolated the gene *CsUp* that might regulate the development of pedicel orientation directly or indirectly in cucumber. Combining the previous study on pedicel orientation and our present result, we speculated that *CsUp* maybe a new type of gene regulating the pedicel orientation in plants.

The CsUp, an Auxilin-like protein, contains a conserved DnaJ domain. Bioinformatics analysis showed that the homologous protein of CsUp, AUXILIN-LIKE7, is also an Auxilin-like protein in *Arabidopsis*. However, the function of AUXILIN-LIKE7 protein is unknown in *Arabidopsis*. In cucumber, the function of six Auxilin-like proteins has not been reported. In *Arabidopsis*, there are seven Auxilin-like proteins, named AUXILIN-LIKE1, AUXILIN-LIKE2, AUXILIN-LIKE3, AUXILIN-LIKE4, AUXILIN-LIKE6, AUXILIN-LIKE7 and auxin-like 1. Both AUXILIN-LIKE1 and AUXILIN-LIKE2 are clathrin uncoating factors involved in clathrin-mediated endocytosis (CME) and overexpression of these factors caused an arrest of growth and development and an inhibition of endocytosis [33]. AUXILIN-LIKE6 involved in phototropin-mediated chloroplast movement in *Arabisopsis* [34]. In rice, *XB21*, an Auxilin-like protein, shows high homology to AUXILIN-LIKE1 and AUXILIN-LIKE2, and plays an important role in plant immune response and cell death regulation [35]. Thus, we speculated that different Auxilin-like proteins have different functions in the same plant and the homologous genes in different plants might play different roles in plant growth. Therefore, we hypothesized that the six Auxilin-like proteins in cucumber may perform different functions in cucumber growth.

A detailed sequence alignment showed that CsUp harbours a conserved domain, named as DnaJ domin (Fig. 4). DnaJ domain plays critical roles in Auxilin-like proteins [36, 37]. Auxilin-like protein is a kind of DnaJ protein and plays an important role in clathrin-mediated endocytosis [38, 39]. In *up*, the 5-bp deletion resulted in the DnaJ domain being lost in the predicted proteins (Fig. 4). In CGN19839, the DnaJ domain was also lost in the predicted proteins caused by the 4-bp deletion (Additional file 5: Figure S5). Furthermore, the A/G transition at exon 1 is far away from the DnaJ domain, thus, this SNP mutation might not inactivate CsUp [37]. These results indicated that the function of CsUp might be destroyed due to the loss of DnaJ domain. The loss-of-function of CsUp caused by DnaJ domain loss might result in the upward-pedicel phenotype in *up* and CGN19839.

We examined the pattern expression of *CsUp* through qRT-PCR (Fig. 6). The results suggested that *CsUp* was strongly expressed in the pedicel of WT and the expression level was much higher than that in *up*. We speculated that the loss-of-function of CsUp would no longer work and formed a feedback inhibition in *up*, which might cause the expression level of *CsUp* much lower than that in WT. In *up*, the pedicels always showed upward growth from the initiation of young fruits to female flowers 5 days after pollination (Fig. 1), and we concluded that the lack of the DnaJ domain might destroy the function of CsUp, and thus the expression of *CsUp* was invariable at different stages in *up*. The results of temporal expression also confirmed our hypothesis (Fig. 7). These results suggested that the CsUp might play an important role in pedicel orientation development in cucumber. However, how CsUp affects the pedicel orientation in cucumber is unclear. Therefore, additional studies could provide glimpses into the regulatory mechanism of pedicel orientation in cucumber.

## Conclusions

The pedicel orientation in *up* mutant is upward growth. *up* is controlled by a single recessive gene. The candidate gene *CsUp* encodes an Auxilin-like protein with the full length of 2613 bp. The mutation, 5-bp deletion in the second exon, of *CsUp* in *up* resulted in a frameshift mutation and earlier translation termination. The identification of *CsUp* may help us to understand the mechanism of pedicel orientation and provide investigation of novel functions of Auxilin-like protein in cucumber.

## Methods

### Plant materials

*up*, a spontaneous mutant possessing upward growth pedicels, was obtained from the wild type 9930. The C-8-6 (WT) is a North China fresh market type inbred line with downward growth pedicels. When the female flowers bloomed, the pedicle of *up* exhibited upward growth with upturned female flowers, while in WT, the pedicel exhibited downward growth with horizontal-facing or downward-facing female flowers (Fig. 1). F_1_ plants were derived from the cross between WT and *up*, then the F_1_ population was self-pollinated to produce the F_2_ population. The F_1_ population was backcrossed with the recessive parent (*up*) to obtain the BC_1_ population. All of the plants were grown under natural sunlight in a greenhouse at Shanghai Jiao Tong University, Shanghai, China.

### InDel and SNP markers development based on genome resequencing

Due to the close genetic relationship and the low polymorphism of SSR markers between *up* and WT (2%), deletion-insertion(InDel)and single nucleotide polymorphism(SNP)markers were developed based on the genome resequencing. The genome of WT was sequenced on the Illumina HiSeq™ 2000 platform (Biomarker Technologies, Beijing, China). With 30-fold sequencing depth, all of the clean reads were mapped to the “9930” genome sequence (http://cucurbitgenomics.org/organism/2, version 2i). InDel and SNP sites between WT and 9930 were detected with the Genious software package. Only fragments with a more than 3 bp insertion or deletion were used to develop InDel markers. For SNP genotyping, approximately 800 bp fragments including the SNP site in the middle of the fragment were amplified and sequenced. The primers were designed with Primer Premier 5.0.

### Phenotypic data collection

The pedicel orientation data of WT, *up*, F_1_ and all individuals from the F_2_ and BC_1_ populations were collected when the female flowers were blooming. The data about pedicel orientation were collected from three to five opening female flowers in each plant.

### DNA extraction and molecular marker analysis

Genomic DNA was extracted from young leaves using the CTAB method [40]. For the SSR and InDel markers, PCR reactions were carried out using a 10 μl volume containing 40 ng genomic DNA, 0.5 μM each primer, 200 μM dNTPs, 1× reaction buffer, and 0.5 U Taq DNA polymerase (Takara Bio Inc., Beijing, China). PCR amplification was performed on a PCR thermocycle instrument (Applied Biosystems, Foster, USA) using the following PCR programme: 94 °C for 5 min; 35 cycles of 94 °C for 30 s, 50–60 °C for 30 s, 72 °C for 30 s; and a final 72 °C for 5 min. Products were separated on the 8% polyacrylamide gel by electrophoresis. After electrophoresis at 220 V for 1.5 h, the gel was separated from the plates and stained in 0.2% AgNO_3_ solution (Shanghai Shi Yi chemicals Reagent, Shanghai, China). Finally, the stained gel was transferred into the developing solution (1.5% sodium hydroxide and 0.4% formaldehyde) to reveal the silver-stained DNA bands. For SNP markers, the PCR reaction and PCR amplification were the same as for the SSR markers. While the reaction samples were 20 μl in volume and the products were analysed by sequencing (Sangon Biotech, Shanghai, China).

### Map-based cloning

The bulked segregation analysis (BSA) method [41] was performed in the F_2_ population to screen for the linkage relationship between markers and the *up* locus. WT and mutant pools (M pool) were constructed by mixing equal amounts of DNA from 10 downward-pedicel and 10 upward-pedicel plants, respectively. Polymorphic SSR markers and InDel markers between C-8-6 and *up* were identified and applied to analyse the WT and M pools. The *up* locus was mapped by using 70 individuals randomly selected from the small F_2_ population (133 individuals). 933 F_2_ individuals (from the small F_2_ population with 133 individuals and the large F_2_ population with 800 individuals) and 524 BC_1_ individuals were used for fine mapping. SSR, InDel and SNP markers, which were used for fine mapping, are listed in Additional file 6: Table S1.

### Candidate gene prediction and gene annotation

Candidate gene prediction was performed using the genome sequence of ‘Gy14’ in Phytozome v10 [42], the genome sequence of ‘9930’ (http://cucurbitgenomics.org/organism/2) [43] and the online program FGENESH (http://www.softberry.com/berry.phtml?topic=fgenesh&group=programs&subgroup=gfind. phtml) [44]. After gene annotation, the corresponding DNA fragments of candidate genes in this region were amplified from *up* and WT using KOD PLUS DNA polymerase (Toyobo, Osaka, Japan) for further sequencing.

### Gene expression analysis by real-time quantitative PCR (qRT-PCR)

We used qRT-PCR to examine the pattern expression of *Csa1G535800* in WT and *up*. Total RNA was extracted from roots, stems, true leaves, female flowers, male flowers, fruits and pedicels from opening female flowers. Reverse transcription was performed by 5× All-In-One RT MasterMix (ABM, Canada). The expression of *Csa1G535800* in pedicels during different periods was also identified. Pedicels from 1 to 1.5 cm length fruits, the young fruits of the day before female flower open and the young fruits with opening female flowers were collected from WT and *up*. In order to exclude *Csa1G535790* and *Csa1G535810*, we also detected their expression in the pedicels during different periods. The Total RNA was extracted by using the method mentioned above. Both the pattern expression and the temporal expression of *Csa1G535800* were identified by using the primer pairs RT-1, which amplify the first exon. The expression of the gene *Csa1G535800* was calculated by the comparative Ct method [45] based on the relative expression of the target gene versus the reference gene *CsActin*. Each test was repeated three times biologically and technically. The primer sequences for qRT-PCR are provided in Additional file 6: Table S1.

### Phylogenetic analysis

Phylogenetic tree was constructed with the homology sequences of CsUP that were downloaded from the NCBI database including the following 10 species: *Arabidopsis thaliana* (Arabidopsis, accession no. NP_174319.1), *Cucumis melo* (melon, accession no. XP_008444679.1), *Actinidia chinensis var. chinensis* (*Actinidia chinensis*, accession no. PSS28916.1), *Glycine max* (soybean, accession no. XP_003535035.3), *Jatropha curcas* (Jatropha, accession no. XP_020535020.1), *Nicotiana tabacum* (tobacco, accession no. XP_016435663.1), *Manihot esculenta* (cassava, accession no. XP_021595454.1), *Solanum lycopersicum* (tomato, accession no. XP_004242447.1), *Oryza sativa Japonica Group* (rice, XP_015617894.1), *Zea mays* (maize, NP_001145320.1) and *Gossypium hirsutum* (cotton, accession no. XP_016688273.1). Protein sequence alignment was accomplished with ClustalW, and the neighbour-joining tree [46] was constructed with the MEGA 7.0 software package (http://www.megasoftware.net/) with 1000 bootstrap replications.

## Additional files

Additional file 1: **Figure S1.** Seven polymorphic markers between WT and the M DNA pool (PDF 71 kb)

Additional file 2: **Figure S2.** Partial results of linkage analysis with indel-*CsUp* demonstrating that this marker is co-segregated with the upward-pedicel phenotype (PDF 91 kb)

Additional file 3: **Figure S3.** Pedicel orientation of B1 and CGN19839 indicating that both inbred lines have the upward-pedicel phenotype (PDF 271 kb)

Additional file 4: **Figure S4.** Structure of Csa1G*535800* and two mutation sites in CGN19839. **a** Structure of *Csa1G535800*. Boxes and lines indicate exons and introns, respectively. Two green boxes in *Csa1G535800* indicate the 5′ UTR and the 3′ UTR. Sequencing results revealed that, compared to WT, CGN19839 has a SNP in the first exon and a 4-bp deletion in the sixth exon of *Csa1G535800*. The yellow box shows the deletion sequence in CGN19839. **b** The position of the SNP and the 4-bp deletion in the encoding sequence. Sequencing results revealed that there was a SNP (‘A’ in C-8-6 and ‘G’ in CGN19839) at 139 bp and a 4-bp deletion at 1234–1237 bp from the ATG start codon (PDF 188 kb)

Additional file 5: **Figure S5.** Alignment of predicted protein sequences between WT and CGN19839. The amino acid highlighted in red is the acid alternation (Ile^47^ in WT to Val^47^ in CGN19839) caused by the SNP in the first exon of *Csa1G535800*. The amino acids highlighted in yellow are abnormal in CGN19839 and the amino acids highlighted in blue are missing in CGN19839. The DnaJ domain at the C-terminal is underlined (PDF 81 kb)

Additional file 6: **Table S1.** Primers used for mapping, cloning and qPCR. (PDF 186 kb) (PDF 185 kb)

Additional file 7: **Figure S6.** Genomic DNA sequence alignment of *CsUP* from WT, *up* and CGN19839 (PDF 231 kb)

Additional file 8: **Figure S7.** Alignment of coding sequence of *CsUP* from WT and *up* with 19 other cucumber lines. The whole coding sequence length is 1404 bp. The 491–495 bp position of *up* and B1 is the 5-bp deletion highlighted in blue. The 139 bp position of CGN19839 that altered from A to G is highlighted in red, and the 1234–1237 bp position in CGN19839 is the 4-bp deletion in blue (PDF 451 kb)

Additional file 9: **Figure S8.** Genomic DNA sequence alignment of *Cs535790* from WT and *up (PDF 67 kb)*

Additional file 10: **Figure S9.** Genomic DNA sequence alignment of *Cs535810* from WT and *up (PDF 43 kb)*

Additional file 11: **Figure S10.** Alignment of promoter sequence of *CsUP* from WT and *up*. (PDF 108 kb)

Additional file 12: **Figure S11.** Alignment of promoter sequence of *Csa1G535790* from WT and *up*. (PDF 109 kb)

Additional file 13: **Figure S12.** Alignment of promoter sequence of *Csa1G535810* from WT and *up*. (PDF 108 kb)

Additional file 14: **Figure S13.** Expression level of *Csa1G535790* and *Csa1G535810* in pedicel at different stages. P1 pedicels from 1 to 1.5 cm length young fruits; P2 pedicels from the young fruits of the day before female flowers open; P3 pedicels from the young fruits with opening female flowers. Data are displayed as the ratio of expression to CsActin3 with three biological replicates. *Error* bars represent standard error (SE) (PDF 133 kb)

## Abbreviations

BSA: Bulked segregation analysis
DAP: Day after pollination
InDel: Insertion-deletion
SNP: Single nucleotide polymorphism
SSR: Simple Sequence Repeats
WT: Wild type
