# Supplementary figures and images for: Mapping and identification of CsUp, a gene encoding an Auxilin-like protein, as a putative candidate gene for the upward-pedicel mutation (up) in cucumber
Source: BMC Plant Biol. 2019 Apr 25;19:157. doi: 10.1186/s12870-019-1772-4 (PMC6485165; doi:10.1186/s12870-019-1772-4)

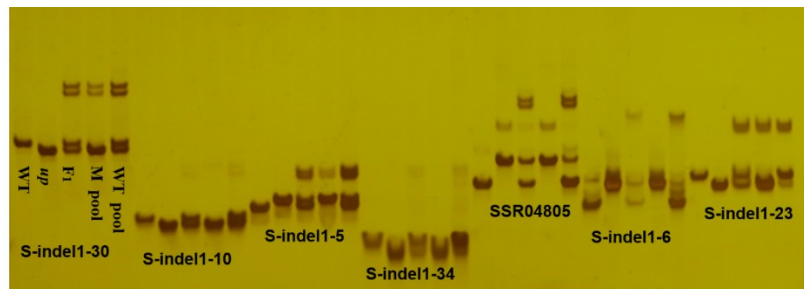

Supplement: Supplementary file 1 — Figure S1. Seven polymorphic markers between WT and the M DNA pool (PDF 71 kb) [file 12870_2019_1772_MOESM1_ESM.pdf]

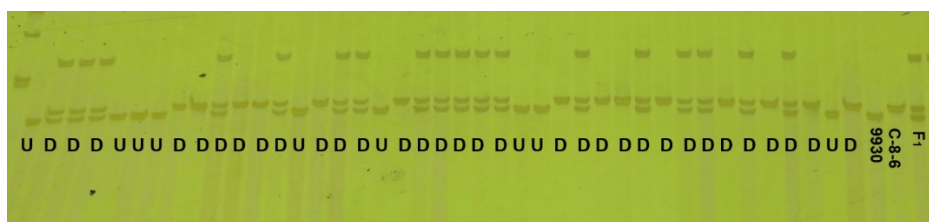

Supplement: Supplementary file 2 — Figure S2. Partial results of linkage analysis with indel-CsUp demonstrating that this marker is co-segregated with the upward-pedicel phenotype (PDF 91 kb) [file 12870_2019_1772_MOESM2_ESM.pdf]

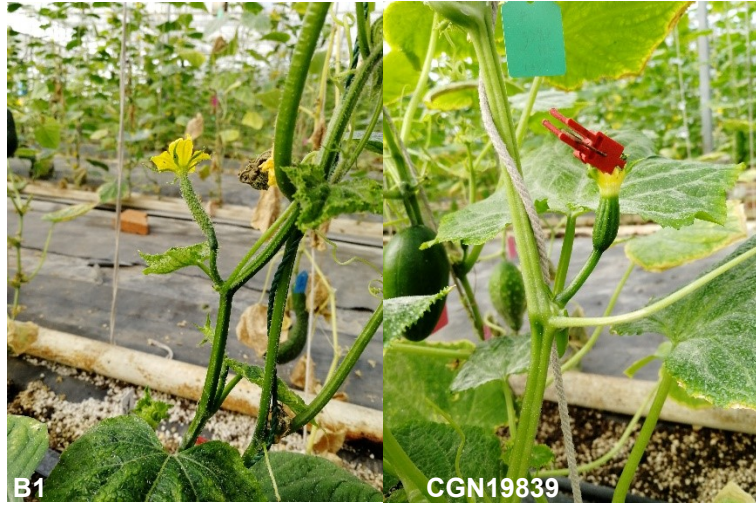

Supplement: Supplementary file 3 — Figure S3. Pedicel orientation of B1 and CGN19839 indicating that both inbred lines have the upward-pedicel phenotype (PDF 271 kb) [file 12870_2019_1772_MOESM3_ESM.pdf]

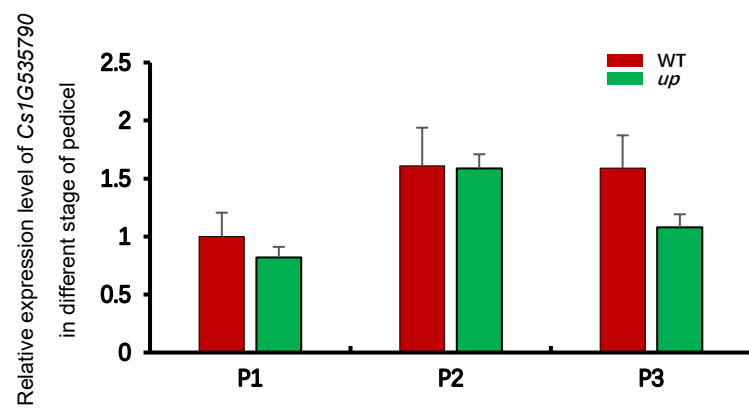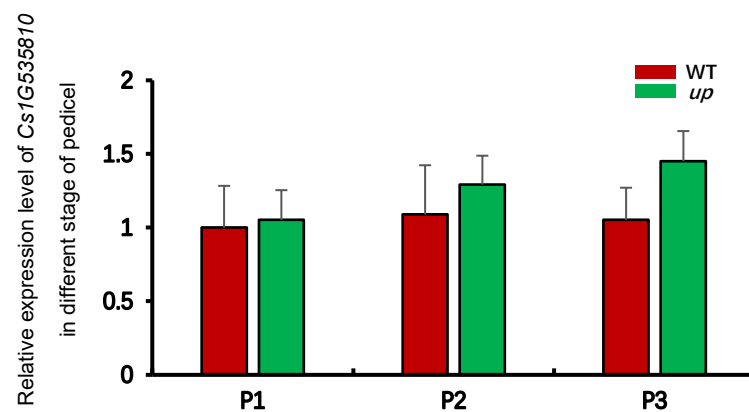

Supplement: Supplementary file 14 — Figure S13. Expression level of Csa1G535790 and Csa1G535810 in pedicel at different stages. P1 pedicels from 1 to 1.5 cm length young fruits; P2 pedicels from the young fruits of the day before female flowers open; P3 pedicels from the young fruits with opening female flowers. Data are displayed as the ratio of expression to CsActin3 with three biological replicates. Error bars represent standard error (SE) (PDF 133 kb) [file 12870_2019_1772_MOESM14_ESM.pdf]
