# Supplementary material for: Mapping and identification of CsUp, a gene encoding an Auxilin-like protein, as a putative candidate gene for the upward-pedicel mutation (up) in cucumber
Source: BMC Plant Biol. 2019 Apr 25;19:157. doi: 10.1186/s12870-019-1772-4 (PMC6485165; doi:10.1186/s12870-019-1772-4)
Supplement: Supplementary file 4 — Figure S4. Structure of Csa1G535800 and two mutation sites in CGN19839. a Structure of Csa1G535800. Boxes and lines indicate exons and introns, respectively. Two green boxes in Csa1G535800 indicate the 5′ UTR and the 3′ UTR. Sequencing results revealed that, compared to WT, CGN19839 has a SNP in the first exon and a 4-bp deletion in the sixth exon of Csa1G535800. The yellow box shows the deletion sequence in CGN19839. b The position of the SNP and the 4-bp deletion in the encoding sequence. Sequencing results revealed that there was a SNP (‘A’ in C-8-6 and ‘G’ in CGN19839) at 139 bp and a 4-bp deletion at 1234–1237 bp from the ATG start codon (PDF 188 kb) [file 12870_2019_1772_MOESM4_ESM.pdf]

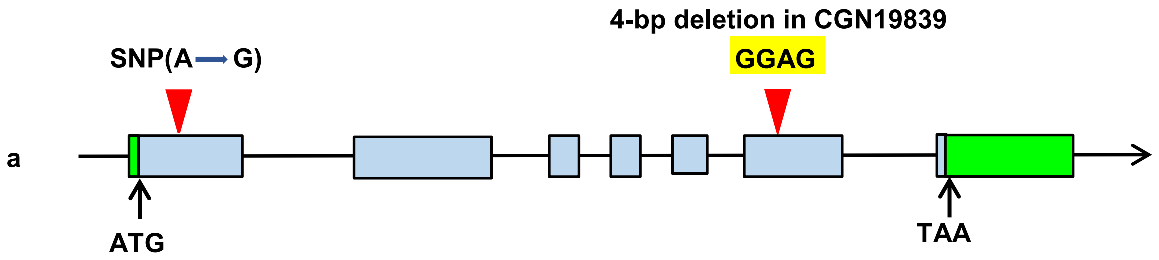

b

```
WT          GGCGGTCCACCACAGACCATTCTCTTCAGGCAATTTTCCGAGAGGTTTGAAGGTATAGAC  180
CGN19839    GGCGGTCCACCACAGACCGTTCTCTTCAGGCAATTTTCCGAGAGGTTTGAAGGTATAGAC  180

WT          GGGTGGTCTCCAATATCGTTGACAAACCTGATTGGAGGCACACAAGTGAAGAAGGCATAT  1260
CGN19839    GGGTGGTCTCCAATATCGTTGACAAACCTGATT----GCACACAAGTGAAGAAGGCATAT  1256
```

Fig. S4 Structure of Csa1G*535800* and two mutation sites in CGN19839

a Structure of *Csa1G535800*. Boxes and lines indicate exons and introns, respectively. Two green boxes in *Csa1G535800* indicate the 5' UTR and the 3' UTR. Sequencing results revealed that, compared to WT, CGN19839 has a SNP in the first exon and a 4-bp deletion in the sixth exon of *Csa1G535800*. The yellow box shows the deletion sequence in CGN19839. **b** The position of the SNP and the 4-bp deletion in the encoding sequence. Sequencing results revealed that there was an SNP ('A' in C-8-6 and 'G' in CGN19839) at 139 bp and a 4-bp deletion at 1234–1237 bp from the ATG start codon.
