# Supplementary material for: Mapping and identification of CsUp, a gene encoding an Auxilin-like protein, as a putative candidate gene for the upward-pedicel mutation (up) in cucumber
Source: BMC Plant Biol. 2019 Apr 25;19:157. doi: 10.1186/s12870-019-1772-4 (PMC6485165; doi:10.1186/s12870-019-1772-4)
Supplement: Supplementary file 5 — Figure S5. Alignment of predicted protein sequences between WT and CGN19839. The amino acid highlighted in red is the acid alternation (Ile47 in WT to Val47 in CGN19839) caused by the SNP in the first exon of Csa1G535800. The amino acids highlighted in yellow are abnormal in CGN19839 and the amino acids highlighted in blue are missing in CGN19839. The DnaJ domain at the C-terminal is underlined (PDF 81 kb) [file 12870_2019_1772_MOESM5_ESM.pdf]

```
WT          MDHTWRLRFGIPRFRSRRSERQTLPKPTSNFLADDFSDVFGGPPQTILFRQFSERFEGID 60
CGN19839    MDHTWRLRFGIPRFRSRRSERQTLPKPTSNFLADDFSDVFGGPPQTVLFRQFSERFEGID 60
            **********************************************:*************
WT          STTSFYEEVFRSSELVSRPQKGGRSLPAFRIPVKEDRFYRDVFGSEDGRRSRDRSEPSSK 120
CGN19839    STTSFYEEVFRSSELVSRPQKGGRSLPAFRIPVKEDRFYRDVFGSEDGRRSRDRSEPSSK 120
            ************************************************************
WT          EFTRSNSSSDFTRLRPVIGDDVAFPSSSSNHRPTNVPTQWNSYTTMFKEQEMPQFAPHLS 180
CGN19839    EFTRSNSSSDFTRLRPVIGDDVAFPSSSSNHRPTNVPTQWNSYTTMFKEQEMPQFAPHLS 180
            ************************************************************
WT          PHMDNRYVEDEYDDRYKSSDHGFGQPVSSPETVILEPNSFRSIKICVDDYLEINSPSSPE 240
CGN19839    PHMDNRYVEDEYDDRYKSSDHGFGQPVSSPETVILEPNSFRSIKICVDDYLEINSPSSPE 240
            ************************************************************
WT          SSLCEDPVYYDGTYCNVLPEDDDDDEDAMSSYVIEITSINREEYREEVSIDEAIAWAKSK 300
CGN19839    SSLCEDPVYYDGTYCNVLPEDDDDDEDAMSSYVIEITSINREEYREEVSIDEAIAWAKSK 300
            ************************************************************
WT          YQSASETDLSVRQQESEQSGEEEGRPVAFECSDQQSNGNGLSQTAETQQREVKVEEEKPQ 360
CGN19839    YQSASETDLSVRQQESEQSGEEEGRPVAFECSDQQSNGNGLSQTAETQQREVKVEEEKPQ 360
            ************************************************************
WT          LNIDRELEGLDEKIKLWSAGKETNIRLLLSTLHYILWSSSGWSPISLTNLIGGTQVKKAY 420
CGN19839    LNIDRELEGLDEKIKLWSAGKETNIRLLLSTLHYILWSSSGWSPISLTNLIAHK------ 414
            ***************************************************. .
WT          QKARLCLHPDKLQQRGATTLQKHVADKAFTILQEAWSVYISQDAFIN 467
CGN19839    ----------------------------------------------- 414
```
