# Supplementary material for: Mapping and identification of CsUp, a gene encoding an Auxilin-like protein, as a putative candidate gene for the upward-pedicel mutation (up) in cucumber
Source: BMC Plant Biol. 2019 Apr 25;19:157. doi: 10.1186/s12870-019-1772-4 (PMC6485165; doi:10.1186/s12870-019-1772-4)
Supplement: Supplementary file 6 — Table S1. Primers used for mapping, cloning and qPCR. (PDF 186 kb) (PDF 185 kb) [file 12870_2019_1772_MOESM6_ESM.pdf]

Table S1. Primers used for mapping, cloning and qPCR

Purposes	Primer name	Chr.	position	Forward primer sequence (5′ to 3′)	Reverse primer sequence (5′ to 3′)	note
**Mapping**	S-indel 1-10	1	10488322	TTTTATTAGTCTATTGTTGGTGAGTTG	GGATTAGCAATGAAATCAAGCA	Indel
	S-indel 1-34	1	16218615	GTCATAATAATAACAATACTTATGGAATC	AACCTAGAGTTAAACTTGAGCAATC	Indel
	S-indel 1-6	1	18610715	ACAAAATAGATAATCAAGCAATGGTA	GAAGACAGGTGATTTCATTTATGTG	Indel
	S-indel 1-23	1	20524790	TTCATTGCTCCCATTTCTCTTC	GGAGGACTAGAAGTTGGAAATACG	Indel
	S-indel 1-66	1	18742186	CAAAATACATCGAAGTTCCCTTTC	GTTCGACCAAGAAGAAAACTATTT	Indel
	S-indel 1-75	1	18927253	CACATCATTTGTGATACGTGGAG	ACATGGTTGATGGTAAAATCCAA	Indel
	S-indel 1-70	1	19712341	AAAAGAAAAAAGTGGTGAAAAAGACA	CTTGGGTTGTGTGGTTAATAAAAG	Indel
	S-SNP 1-31	1	19033609	ATTTTGGGCTTCCGTTAGAAA	CTTGGTGCTTTCCTTTCCATT	SNP
	S-SNP 1-35	1	19188768	TTCTACTCTTCTCCTTTTCATCATTTC	TTTTCCAAACCAATTTCCAGG	SNP
	S-SNP 1-37	1	19201994	ATTCTTAAAAGCATTTCCACATTG	TGTTTTGTTTGGGTGAGATGAG	SNP
	S-SNP 1-40	1	19224010	ATCATTCTCTTATTTAGCCAACGC	TATCATCCTCGGTTTACAATAGACTAC	SNP
	S-SNP 1-28	1	19240215	AATCCTCATCTCTTAATCCCCAC	CCACCCAAGCTTTACAAGTTGTA	SNP
	S-SNP 1-11	1	19297029	TAACGAGTAGGGGAAAATCAACATA	AGTGTTCTTTTTCCACCATTTCTAA	SNP
	S-SNP 1-51	1	19305601	GTACCTCCCAATAGAAAGTTAAACG	AGAAGTAACGAATCGTCTGCAATA	SNP
	S-SNP 1-53	1	19315520	TGGATTAAATGATGGAGTAGGAGTG	AACTCTTACTTGGGATACTTCAAAACA	SNP
	S-SNP 1-12	1	19335763	TGCCACACGCAACAAGTTAAT	GAAAGTAAATAAAGGGTAGGTAGTCAAG	SNP
	S-SNP 1-13	1	19364247	AGTTAGGTTTCGTTGTTGACATTG	AAAGATCACACGTCACACCCC	SNP
	S-SNP 1-7	1	19511180	CAACAGGACGATTCATGTTTCTAC	TGGAGCTGATCCTTAGGTGTGT	SNP
	Indel-CsUp			AACCAATGTCCCAACACAATG	GGGAGAGATGAGGTGCAAACT	Indel
DNA cloning	Csa1G535790			ATGGGTATGTCAACTAACTACTGTAAGG	GCTTTATTTAATATCCAAACGTGGTA	
	Csa1G535800			TCTCATTCTCTCTCACTCTCTCACTC	CAACAGAGGCATTGTGATTGACT	
	Csa1G535810			AATTTAGTTTCCAACCAAACCAGT	AGTTGTACCTGAATAACTCAGGCTACC	
	Indel-CsUp			AACCAATGTCCCAACACAATG	GGGAGAGATGAGGTGCAAACT	
cDNA cloning	cds535800			TCCCTCTTTTGTCTCCCCC	ATGGGTATCAGCTCGTGAATGT	
qPCR	RT-1			ACCATACTTGGCGTCTCCGT	ATACCTTCAAACCTCTCGGAAAA	
	CsActin3			TCGTGCTGGATTCTGGTG	GGCAGTGGTGGTGAACAT	
