# Supplementary material for: Mapping and identification of CsUp, a gene encoding an Auxilin-like protein, as a putative candidate gene for the upward-pedicel mutation (up) in cucumber
Source: BMC Plant Biol. 2019 Apr 25;19:157. doi: 10.1186/s12870-019-1772-4 (PMC6485165; doi:10.1186/s12870-019-1772-4)
Supplement: Supplementary file 7 — Figure S6. Genomic DNA sequence alignment of CsUP from WT, up and CGN19839 (PDF 231 kb) [file 12870_2019_1772_MOESM7_ESM.pdf]

```
WT          ATGGACCATACTTGGCGTCTCCGTTTTGGAATTCCCCGTTTCCGTTCCCGGAGATCCGAA    60
up          ATGGACCATACTTGGCGTCTCCGTTTTGGAATTCCCCGTTTCCGTTCCCGGAGATCCGAA    60
CGN19839    ATGGACCATACTTGGCGTCTCCGTTTTGGAATTCCCCGTTTCCGTTCCCGGAGATCCGAA    60
            ************************************************************

WT          CGCCAAACCCTCCCCAAACCCACTTCTAATTTCCTCGCCGACGACTTCTCCGACGTCTTC    120
up          CGCCAAACCCTCCCCAAACCCACTTCTAATTTCCTCGCCGACGACTTCTCCGACGTCTTC    120
CGN19839    CGCCAAACCCTCCCCAAACCCACTTCTAATTTCCTCGCCGACGACTTCTCCGACGTCTTC    120
            ************************************************************

WT          GGCGGTCCACCACAGACCATTCTCTTCAGGCAATTTTCCGAGAGGTTTGAAGGTATAGAC    180
up          GGCGGTCCACCACAGACCATTCTCTTCAGGCAATTTTCCGAGAGGTTTGAAGGTATAGAC    180
CGN19839    GGCGGTCCACCACAGACCGTTCTCTTCAGGCAATTTTCCGAGAGGTTTGAAGGTATAGAC    180
            ******************.*****************************************

WT          TCTACTACTTCATTCTACGAAGAAGTATTCCGCTCCTCCGAGCTAGTTTCCCGACCGCAG    240
up          TCTACTACTTCATTCTACGAAGAAGTATTCCGCTCCTCCGAGCTAGTTTCCCGACCGCAG    240
CGN19839    TCTACTACTTCATTCTACGAAGAAGTATTCCGCTCCTCCGAGCTAGTTTCCCGACCGCAG    240
            ************************************************************

WT          AAGGGTGGCCGGAGCTTGCCTGCCTTTAGAATCCCTGTTAAGGAGGATAGATTTTACCGC    300
up          AAGGGTGGCCGGAGCTTGCCTGCCTTTAGAATCCCTGTTAAGGAGGATAGATTTTACCGC    300
CGN19839    AAGGGTGGCCGGAGCTTGCCTGCCTTTAGAATCCCTGTTAAGGAGGATAGATTTTACCGC    300
            ************************************************************

WT          GATGTTTTTGGATCGGAAGATGGTCGACGGTCGAGAGATAGGTCGGAGCCGAGCTCTAAG    360
up          GATGTTTTTGGATCGGAAGATGGTCGACGGTCGAGAGATAGGTCGGAGCCGAGCTCTAAG    360
CGN19839    GATGTTTTTGGATCGGAAGATGGTCGACGGTCGAGAGATAGGTCGGAGCCGAGCTCTAAG    360
            ************************************************************

WT          GAATTCACTAGATCGAACTCGTCTTCTGATTTCACCCGTCTTCGGCCGGTCATCGGAGAT    420
up          GAATTCACTAGATCGAACTCGTCTTCTGATTTCACCCGTCTTCGGCCGGTCATCGGAGAT    420
CGN19839    GAATTCACTAGATCGAACTCGTCTTCTGATTTCACCCGTCTTCGGCCGGTCATCGGAGAT    420
            ************************************************************

WT          GACGTGGCGTTCCCTTCGTCGTCTTCAAATCACAGGTTGGTAGTGTACTGCAGAAAGTGA    480
up          GACGTGGCGTTCCCTTCGTCGTCTTCAAATCACAGGTTGGTAGTGTACTGCAGAAAGTGA    480
CGN19839    GACGTGGCGTTCCCTTCGTCGTCTTCAAATCACAGGTTGGTAGTGTACTGCAGAAAGTGA    480
            ************************************************************

WT          GGGGGCCAAACCCTTATTTTGGATTATTCTATTGGACATGTATGCCCTCTATGGCTTAGG    540
up          GGGGGCCAAACCCTTATTTTGGATTATTCTATTGGACATGTATGCCCTCTATGGCTTAGG    540
CGN19839    GGGGGCCAAACCCTTATTTTGGATTATTCTATTGGACATGTATGCCCTCTATGGCTTAGG    540
            ************************************************************

WT          TCATTTTTCATATGTTGTTTGATATTGTTTGGCTGTTTCATGAGGGTATTTAAGAGAAAT    600
up          TCATTTTTCATATGTTGTTTGATATTGTTTGGCTGTTTCATGAGGGTATTTAAGAGAAAT    600
CGN19839    TCATTTTTCATATGTTGTTTGATATTGTTTGGCTGTTTCATGAGGGTATTTAAGAGAAAT    600
            ************************************************************
```

```
WT          GATAATCTTAATCAGTCTGATTCATAGTAATATGTTTATTGATACTAACAACCAATTTTG    660
up          GATAATCTTAATCAGTCTGATTCATAGTAATATGTTTATTGATACTAACAACCAATTTTG    660
CGN19839    GATAATCTTAATCAGTCTGATTCATAGTAATATGTTTATTGATACTAACAACCAATTTTG    660
            ************************************************************

WT          TTTTATTGATGGCTTTTCTTAAGCATGAATGTTTGATTATAAATCTGTCTCTAAATTGAT    720
up          TTTTATTGATGGCTTTTCTTAAGCATGAATGTTTGATTATAAATCTGTCTCTAAATTGAT    720
CGN19839    TTTTATTGATGGCTTTTCTTAAGCATGAATGTTTGATTATAAATCTGTCTCTAAATTGAT    720
            ************************************************************

WT          TCTTTTTTTAAACTTGATTTGACCAGCATTGTCTAACACTTAATTACTTTAATGTGATTT    780
up          TCTTTTTTTAAACTTGATTTGACCAGCATTGTCTAACACTTAATTACTTTAATGTGATTT    780
CGN19839    TCTTTTTTTAAACTTGATTTGACCAGCATTGTCTAACACTTAATTACTTTAATGTGATTT    780
            ************************************************************

WT          CAATATTCATTTCCTTATTTTAGTTTGGTTGCAAGTATTCAATATCTATCTCTTCAATTC    840
up          CAATATTCATTTCCTTATTTTAGTTTGGTTGCAAGTATTCAATATCTATCTCTTCAATTC    840
CGN19839    CAATATTCATTTCCTTATTTTAGTTTGGTTGCAAGTATTCAATATCTATCTCTTCAATTC    840
            ************************************************************

WT          ATATAGGCCAACCAATGTCCCAACACAATGGAATTCATACACAACTATGTTCAAGGAACA    900
up          ATATAGGCCAACCAATGTCCCAACACAATGGAATTCATACA-----ATGTTCAAGGAACA    895
CGN19839    ATATAGGCCAACCAATGTCCCAACACAATGGAATTCATACACAACTATGTTCAAGGAACA    900
            ****************************************      **************

WT          GGAAATGCCACAGTTTGCACCTCATCTCTCCCCTCATATGGATAACCGTTATGTAGAAGA    960
up          GGAAATGCCACAGTTTGCACCTCATCTCTCCCCTCATATGGATAACCGTTATGTAGAAGA    955
CGN19839    GGAAATGCCACAGTTTGCACCTCATCTCTCCCCTCATATGGATAACCGTTATGTAGAAGA    960
            ************************************************************

WT          TGAATATGATGATAGATACAAAAGCTCAGACCATGGATTTGGACAGCCTGTATCATCGCC    1020
up          TGAATATGATGATAGATACAAAAGCTCAGACCATGGATTTGGACAGCCTGTATCATCGCC    1015
CGN19839    TGAATATGATGATAGATACAAAAGCTCAGACCATGGATTTGGACAGCCTGTATCATCGCC    1020
            ************************************************************

WT          AGAAACCGTTATTCTGGAACCAAATTCGTTCAGAAGCATCAAGATCTGCGTGGATGATTA    1080
up          AGAAACCGTTATTCTGGAACCAAATTCGTTCAGAAGCATCAAGATCTGCGTGGATGATTA    1075
CGN19839    AGAAACCGTTATTCTGGAACCAAATTCGTTCAGAAGCATCAAGATCTGCGTGGATGATTA    1080
            ************************************************************

WT          TTTAGAAATAAACTCCCCATCATCTCCTGAATCTTCTCTCTGTGAGGATCCAGTTTATTA    1140
up          TTTAGAAATAAACTCCCCATCATCTCCTGAATCTTCTCTCTGTGAGGATCCAGTTTATTA    1135
CGN19839    TTTAGAAATAAACTCCCCATCATCTCCTGAATCTTCTCTCTGTGAGGATCCAGTTTATTA    1140
            ************************************************************

WT          TGATGGAACTTACTGTAATGTTTTACCGGAAGATGACGATGACGATGAAGATGCTATGAG    1200
up          TGATGGAACTTACTGTAATGTTTTACCGGAAGATGACGATGACGATGAAGATGCTATGAG    1195
CGN19839    TGATGGAACTTACTGTAATGTTTTACCGGAAGATGACGATGACGATGAAGATGCTATGAG    1200
            ************************************************************
```

```
WT          CTCTTATGTCATTGAGATAACTTCTATCAATAGAGAAGAATATAGAGAAGAAGTTTCTAT    1260
up          CTCTTATGTCATTGAGATAACTTCTATCAATAGAGAAGAATATAGAGAAGAAGTTTCTAT    1255
CGN19839    CTCTTATGTCATTGAGATAACTTCTATCAATAGAGAAGAATATAGAGAAGAAGTTTCTAT    1260
            ************************************************************

WT          TGACGAAGCAATTGCTTGGGCTAAATCGAAGTATCAAAGTGCATCTGAGACAGATTTGAG    1320
up          TGACGAAGCAATTGCTTGGGCTAAATCGAAGTATCAAAGTGCATCTGAGACAGATTTGAG    1315
CGN19839    TGACGAAGCAATTGCTTGGGCTAAATCGAAGTATCAAAGTGCATCTGAGACAGATTTGAG    1320
            ************************************************************

WT          TGTTAGACAACAAGAAAGCGAGCAATCTGGGGAAGAAGAAGGTAAACTTTCATTAACTCC    1380
up          TGTTAGACAACAAGAAAGCGAGCAATCTGGGGAAGAAGAAGGTAAACTTTCATTAACTCC    1375
CGN19839    TGTTAGACAACAAGAAAGCGAGCAATCTGGGGAAGAAGAAGGTAAACTTTCATTAACTCC    1380
            ************************************************************

WT          ACGTGTGTTTCATGCTGGCCGGACCTGTTTTGTATGAATGTTTAACCTCCATAGTGAAAA    1440
up          ACGTGTGTTTCATGCTGGCCGGACCTGTTTTGTATGAATGTTTAACCTCCATAGTGAAAA    1435
CGN19839    ACGTGTGTTTCATGCTGGCCGGACCTGTTTTGTATGAATGTTTAACCTCCATAGTGAAAA    1440
            ************************************************************

WT          TTCTAAAAGTATTCTTCTTTTTTTGTTTTGAAGGAAGACCTGTTGCATTTGAATGCTCAG    1500
up          TTCTAAAAGTATTCTTCTTTTTTTGTTTTGAAGGAAGACCTGTTGCATTTGAATGCTCAG    1495
CGN19839    TTCTAAAAGTATTCTTCTTTTTTTGTTTTGAAGGAAGACCTGTTGCATTTGAATGCTCAG    1500
            ************************************************************

WT          ATCAGCAGTCGAATGGAAATGGATTGTCGCAGACTGCAGAGGTAACAATTTTCTACTTTT    1560
up          ATCAGCAGTCGAATGGAAATGGATTGTCGCAGACTGCAGAGGTAACAATTTTCTACTTTT    1555
CGN19839    ATCAGCAGTCGAATGGAAATGGATTGTCGCAGACTGCAGAGGTAACAATTTTCTACTTTT    1560
            ************************************************************

WT          CACATAAAATCTGAAATAGGAAAACTCTTTACGCCATTCATCTCAATACTGGTCAGAAAA    1620
up          CACATAAAATCTGAAATAGGAAAACTCTTTACGCCATTCATCTCAATACTGGTCAGAAAA    1615
CGN19839    CACATAAAATCTGAAATAGGAAAACTCTTTACGCCATTCATCTCAATACTGGTCAGAAAA    1620
            ************************************************************

WT          CATTCTGGATAATGCTTAGAACTTGGAAGAATCTATGAATCTAATTGATTGAATATCTTT    1680
up          CATTCTGGATAATGCTTAGAACTTGGAAGAATCTATGAATCTAATTGATTGAATATCTTT    1675
CGN19839    CATTCTGGATAATGCTTAGAACTTGGAAGAATCTATGAATCTAATTGATTGAATATCTTT    1680
            ************************************************************

WT          TGCAATTGTGTAATAAAGCTGAATTGCGTTGCTTAACTGGGTGCTCTTGTTTCTGCCTCA    1740
up          TGCAATTGTGTAATAAAGCTGAATTGCGTTGCTTAACTGGGTGCTCTTGTTTCTGCCTCA    1735
CGN19839    TGCAATTGTGTAATAAAGCTGAATTGCGTTGCTTAACTGGGTGCTCTTGTTTCTGCCTCA    1740
            ************************************************************

WT          GACACAACAGAGAGAAGTAAAAGTTGAAGAAGAAAAGCCACAGTTGAACATCGATGTAAG    1800
up          GACACAACAGAGAGAAGTAAAAGTTGAAGAAGAAAAGCCACAGTTGAACATCGATGTAAG    1795
CGN19839    GACACAACAGAGAGAAGTAAAAGTTGAAGAAGAAAAGCCACAGTTGAACATCGATGTAAG    1800
            ************************************************************
```

```
WT          TAGATTATCACAGGGCTTTATATTTTTAAGCTTGGCACTATTTCATTTCCCTCGAGTTTT    1860
up          TAGATTATCACAGGGCTTTATATTTTTAAGCTTGGCACTATTTCATTTCCCTCGAGTTTT    1855
CGN19839    TAGATTATCACAGGGCTTTATATTTTTAAGCTTGGCACTATTTCATTTCCCTCGAGTTTT    1860
            ************************************************************

WT          GAAGTATAAGATATTTTCATGGAATGGCTCCTACTGGTAGTTCCAAATTTAGGAAAAAGA    1920
up          GAAGTATAAGATATTTTCATGGAATGGCTCCTACTGGTAGTTCCAAATTTAGGAAAAAGA    1915
CGN19839    GAAGTATAAGATATTTTCATGGAATGGCTCCTACTGGTAGTTCCAAATTTAGGAAAAAGA    1920
            ************************************************************

WT          GCAATCAAGCTAATTAATTAACACAAAATCATACAAAGTGTTCATTTTATTTCTGGCTAA    1980
up          GCAATCAAGCTAATTAATTAACACAAAATCATACAAAGTGTTCATTTTATTTCTGGCTAA    1975
CGN19839    GCAATCAAGCTAATTAATTAACACAAAATCATACAAAGTGTTCATTTTATTTCTGGCTAA    1980
            ************************************************************

WT          GGAACAAGTTTTTGATCTCTGCTTTTTTCTCCTTATCTCAAATAACAGAGAGAATTGGAA    2040
up          GGAACAAGTTTTTGATCTCTGCTTTTTTCTCCTTATCTCAAATAACAGAGAGAATTGGAA    2035
CGN19839    GGAACAAGTTTTTGATCTCTGCTTTTTTCTCCTTATCTCAAATAACAGAGAGAATTGGAA    2040
            ************************************************************

WT          GGATTAGATGAAAAAAATAAAGCTATGGTCAGCTGGCAAGGAGACCAACATCCGCTTGCTA    2100
up          GGATTAGATGAAAAAAATAAAGCTATGGTCAGCTGGCAAGGAGACCAACATCCGCTTGCTA    2095
CGN19839    GGATTAGATGAAAAAAATAAAGCTATGGTCAGCTGGCAAGGAGACCAACATCCGCTTGCTA    2100
            ************************************************************

WT          CTTTCTACACTTCATTATGTAAGTTCCTTCATGCCAGATAACATCATGGACATCTCCCTC    2160
up          CTTTCTACACTTCATTATGTAAGTTCCTTCATGCCAGATAACATCATGGACATCTCCCTC    2155
CGN19839    CTTTCTACACTTCATTATGTAAGTTCCTTCATGCCAGATAACATCATGGACATCTCCCTC    2160
            ************************************************************

WT          CTTTTCATGCTATGAAACTCCAATCTAACGATCCAAAACCATTTCATCTGGGATTATTAC    2220
up          CTTTTCATGCTATGAAACTCCAATCTAACGATCCAAAACCATTTCATCTGGGATTATTAC    2215
CGN19839    CTTTTCATGCTATGAAACTCCAATCTAACGATCCAAAACCATTTCATCTGGGATTATTAC    2220
            ************************************************************

WT          AGATATTGTGGTCAAGTAGTGGGTGGTCTCCAATATCGTTGACAAACCTGATTGGAGGCA    2280
up          AGATATTGTGGTCAAGTAGTGGGTGGTCTCCAATATCGTTGACAAACCTGATTGGAGGCA    2275
CGN19839    AGATATTGTGGTCAAGTAGTGGGTGGTCTCCAATATCGTTGACAAACCTGATT----GCA    2276
            *****************************************************    ***

WT          CACAAGTGAAGAAGGCATATCAAAAAGCAAGATTATGTCTCCACCCAGATAAGCTGCAGC    2340
up          CACAAGTGAAGAAGGCATATCAAAAAGCAAGATTATGTCTCCACCCAGATAAGCTGCAGC    2335
CGN19839    CACAAGTGAAGAAGGCATATCAAAAAGCAAGATTATGTCTCCACCCAGATAAGCTGCAGC    2336
            ************************************************************

WT          AAAGAGGAGCGACAACGCTGCAGAAACATGTTGCGGATAAGGCTTTTACCATCCTTCAGG    2400
up          AAAGAGGAGCGACAACGCTGCAGAAACATGTTGCGGATAAGGCTTTTACCATCCTTCAGG    2395
CGN19839    AAAGAGGAGCGACAACGCTGCAGAAACATGTTGCGGATAAGGCTTTTACCATCCTTCAGG    2396
            ************************************************************
```

```
WT          TAACTCTTCGCTCATACACAATATTTAATGACCATCTGATTCAACCAAGAACTTGAAGTC    2460
up          TAACTCTTCGCTCATACACAATATTTAATGACCATCTGATTCAACCAAGAACTTGAAGTC    2455
CGN19839    TAACTCTTCGCTCATACACAATATTTAATGACCATCTGATTCAACCAAGAACTTGAAGTC    2456
            ************************************************************

WT          AAACACTAGATAAAAAATGTAACTAATAAGACTGAATTTGTGAGAAAAATGTTGAATAGT    2520
up          AAACACTAGATAAAAAATGTAACTAATAAGACTGAATTTGTGAGAAAAATGTTGAATAGT    2515
CGN19839    AAACACTAGATAAAAAATGTAACTAATAAGACTGAATTTGTGAGAAAAATGTTGAATAGT    2516
            ************************************************************

WT          ATGTTTATTTGCAAGATTTTGATTTTTATGTCTTATTGTGTTTTACAGGAAGCGTGGTCT    2580
up          ATGTTTATTTGCAAGATTTTGATTTTTATGTCTTATTGTGTTTTACAGGAAGCGTGGTCT    2575
CGN19839    ATGTTTATTTGCAAGATTTTGATTTTTATGTCTTATTGTGTTTTACAGGAAGCGTGGTCT    2576
            ************************************************************

WT          GTATATATATCTCAAGATGCCTTCATCAACTAA 2613
up          GTATATATATCTCAAGATGCCTTCATCAACTAA 2608
CGN19839    GTATATATATCTCAAGATGCCTTCATCAACTAA 2609
            *********************************
```
