# Supplementary material for: Mapping and identification of CsUp, a gene encoding an Auxilin-like protein, as a putative candidate gene for the upward-pedicel mutation (up) in cucumber
Source: BMC Plant Biol. 2019 Apr 25;19:157. doi: 10.1186/s12870-019-1772-4 (PMC6485165; doi:10.1186/s12870-019-1772-4)
Supplement: Supplementary file 8 — Figure S7. Alignment of coding sequence of CsUP from WT and up with 19 other cucumber lines. The whole coding sequence length is 1404 bp. The 491–495 bp position of up and B1 is the 5-bp deletion highlighted in blue. The 139 bp position of CGN19839 that altered from A to G is highlighted in red, and the 1234–1237 bp position in CGN19839 is the 4-bp deletion in blue (PDF 451 kb) [file 12870_2019_1772_MOESM8_ESM.pdf]

```
WT          ATGGACCATACTTGGCGTCTCCGTTTTGGAATTCCCCGTTTCCGTTCCCGGAGATCCGAA   60
9930        ATGGACCATACTTGGCGTCTCCGTTTTGGAATTCCCCGTTTCCGTTCCCGGAGATCCGAA   60
up          ATGGACCATACTTGGCGTCTCCGTTTTGGAATTCCCCGTTTCCGTTCCCGGAGATCCGAA   60
B1          ATGGACCATACTTGGCGTCTCCGTTTTGGAATTCCCCGTTTCCGTTCCCGGAGATCCGAA   60
CGN19839    ATGGACCATACTTGGCGTCTCCGTTTTGGAATTCCCCGTTTCCGTTCCCGGAGATCCGAA   60
30          ATGGACCATACTTGGCGTCTCCGTTTTGGAATTCCCCGTTTCCGTTCCCGGAGATCCGAA   60
61          ATGGACCATACTTGGCGTCTCCGTTTTGGAATTCCCCGTTTCCGTTCCCGGAGATCCGAA   60
422         ATGGACCATACTTGGCGTCTCCGTTTTGGAATTCCCCGTTTCCGTTCCCGGAGATCCGAA   60
L02         ATGGACCATACTTGGCGTCTCCGTTTTGGAATTCCCCGTTTCCGTTCCCGGAGATCCGAA   60
L03         ATGGACCATACTTGGCGTCTCCGTTTTGGAATTCCCCGTTTCCGTTCCCGGAGATCCGAA   60
S1003       ATGGACCATACTTGGCGTCTCCGTTTTGGAATTCCCCGTTTCCGTTCCCGGAGATCCGAA   60
S05         ATGGACCATACTTGGCGTCTCCGTTTTGGAATTCCCCGTTTCCGTTCCCGGAGATCCGAA   60
S06         ATGGACCATACTTGGCGTCTCCGTTTTGGAATTCCCCGTTTCCGTTCCCGGAGATCCGAA   60
WX2         ATGGACCATACTTGGCGTCTCCGTTTTGGAATTCCCCGTTTCCGTTCCCGGAGATCCGAA   60
S52         ATGGACCATACTTGGCGTCTCCGTTTTGGAATTCCCCGTTTCCGTTCCCGGAGATCCGAA   60
S94         ATGGACCATACTTGGCGTCTCCGTTTTGGAATTCCCCGTTTCCGTTCCCGGAGATCCGAA   60
SB-2        ATGGACCATACTTGGCGTCTCCGTTTTGGAATTCCCCGTTTCCGTTCCCGGAGATCCGAA   60
SD4         ATGGACCATACTTGGCGTCTCCGTTTTGGAATTCCCCGTTTCCGTTCCCGGAGATCCGAA   60
WD1         ATGGACCATACTTGGCGTCTCCGTTTTGGAATTCCCCGTTTCCGTTCCCGGAGATCCGAA   60
WD2         ATGGACCATACTTGGCGTCTCCGTTTTGGAATTCCCCGTTTCCGTTCCCGGAGATCCGAA   60
WX1         ATGGACCATACTTGGCGTCTCCGTTTTGGAATTCCCCGTTTCCGTTCCCGGAGATCCGAA   60
WZ1         ATGGACCATACTTGGCGTCTCCGTTTTGGAATTCCCCGTTTCCGTTCCCGGAGATCCGAA   60
            ************************************************************
WT          CGCCAAACCCTCCCCAAACCCACTTCTAATTTCCTCGCCGACGACTTCTCCGACGTCTTC   120
9930        CGCCAAACCCTCCCCAAACCCACTTCTAATTTCCTCGCCGACGACTTCTCCGACGTCTTC   120
up          CGCCAAACCCTCCCCAAACCCACTTCTAATTTCCTCGCCGACGACTTCTCCGACGTCTTC   120
B1          CGCCAAACCCTCCCCAAACCCACTTCTAATTTCCTCGCCGACGACTTCTCCGACGTCTTC   120
CGN19839    CGCCAAACCCTCCCCAAACCCACTTCTAATTTCCTCGCCGACGACTTCTCCGACGTCTTC   120
30          CGCCAAACCCTCCCCAAACCCACTTCTAATTTCCTCGCCGACGACTTCTCCGACGTCTTC   120
61          CGCCAAACCCTCCCCAAACCCACTTCTAATTTCCTCGCCGACGACTTCTCCGACGTCTTC   120
422         CGCCAAACCCTCCCCAAACCCACTTCTAATTTCCTCGCCGACGACTTCTCCGACGTCTTC   120
L02         CGCCAAACCCTCCCCAAACCCACTTCTAATTTCCTCGCCGACGACTTCTCCGACGTCTTC   120
L03         CGCCAAACCCTCCCCAAACCCACTTCTAATTTCCTCGCCGACGACTTCTCCGACGTCTTC   120
S1003       CGCCAAACCCTCCCCAAACCCACTTCTAATTTCCTCGCCGACGACTTCTCCGACGTCTTC   120
S05         CGCCAAACCCTCCCCAAACCCACTTCTAATTTCCTCGCCGACGACTTCTCCGACGTCTTC   120
S06         CGCCAAACCCTCCCCAAACCCACTTCTAATTTCCTCGCCGACGACTTCTCCGACGTCTTC   120
WX2         CGCCAAACCCTCCCCAAACCCACTTCTAATTTCCTCGCCGACGACTTCTCCGACGTCTTC   120
S52         CGCCAAACCCTCCCCAAACCCACTTCTAATTTCCTCGCCGACGACTTCTCCGACGTCTTC   120
S94         CGCCAAACCCTCCCCAAACCCACTTCTAATTTCCTCGCCGACGACTTCTCCGACGTCTTC   120
SB-2        CGCCAAACCCTCCCCAAACCCACTTCTAATTTCCTCGCCGACGACTTCTCCGACGTCTTC   120
SD4         CGCCAAACCCTCCCCAAACCCACTTCTAATTTCCTCGCCGACGACTTCTCCGACGTCTTC   120
WD1         CGCCAAACCCTCCCCAAACCCACTTCTAATTTCCTCGCCGACGACTTCTCCGACGTCTTC   120
WD2         CGCCAAACCCTCCCCAAACCCACTTCTAATTTCCTCGCCGACGACTTCTCCGACGTCTTC   120
WX1         CGCCAAACCCTCCCCAAACCCACTTCTAATTTCCTCGCCGACGACTTCTCCGACGTCTTC   120
WZ1         CGCCAAACCCTCCCCAAACCCACTTCTAATTTCCTCGCCGACGACTTCTCCGACGTCTTC   120
            ************************************************************
```

```
WT          GGCGGTCCACCACAGACCATTCTCTTCAGGCAATTTTCCGAGAGGTTTGAAGGTATAGAC   180
9930        GGCGGTCCACCACAGACCATTCTCTTCAGGCAATTTTCCGAGAGGTTTGAAGGTATAGAC   180
up          GGCGGTCCACCACAGACCATTCTCTTCAGGCAATTTTCCGAGAGGTTTGAAGGTATAGAC   180
B1          GGCGGTCCACCACAGACCATTCTCTTCAGGCAATTTTCCGAGAGGTTTGAAGGTATAGAC   180
CGN19839    GGCGGTCCACCACAGACCGTTCTCTTCAGGCAATTTTCCGAGAGGTTTGAAGGTATAGAC   180
30          GGCGGTCCACCACAGACCATTCTCTTCAGGCAATTTTCCGAGAGGTTTGAAGGTATAGAC   180
61          GGCGGTCCACCACAGACCATTCTCTTCAGGCAATTTTCCGAGAGGTTTGAAGGTATAGAC   180
422         GGCGGTCCACCACAGACCATTCTCTTCAGGCAATTTTCCGAGAGGTTTGAAGGTATAGAC   180
L02         GGCGGTCCACCACAGACCATTCTCTTCAGGCAATTTTCCGAGAGGTTTGAAGGTATAGAC   180
L03         GGCGGTCCACCACAGACCATTCTCTTCAGGCAATTTTCCGAGAGGTTTGAAGGTATAGAC   180
S1003       GGCGGTCCACCACAGACCATTCTCTTCAGGCAATTTTCCGAGAGGTTTGAAGGTATAGAC   180
S05         GGCGGTCCACCACAGACCATTCTCTTCAGGCAATTTTCCGAGAGGTTTGAAGGTATAGAC   180
S06         GGCGGTCCACCACAGACCATTCTCTTCAGGCAATTTTCCGAGAGGTTTGAAGGTATAGAC   180
WX2         GGCGGTCCACCACAGACCATTCTCTTCAGGCAATTTTCCGAGAGGTTTGAAGGTATAGAC   180
S52         GGCGGTCCACCACAGACCATTCTCTTCAGGCAATTTTCCGAGAGGTTTGAAGGTATAGAC   180
S94         GGCGGTCCACCACAGACCATTCTCTTCAGGCAATTTTCCGAGAGGTTTGAAGGTATAGAC   180
SB-2        GGCGGTCCACCACAGACCATTCTCTTCAGGCAATTTTCCGAGAGGTTTGAAGGTATAGAC   180
SD4         GGCGGTCCACCACAGACCATTCTCTTCAGGCAATTTTCCGAGAGGTTTGAAGGTATAGAC   180
WD1         GGCGGTCCACCACAGACCATTCTCTTCAGGCAATTTTCCGAGAGGTTTGAAGGTATAGAC   180
WD2         GGCGGTCCACCACAGACCATTCTCTTCAGGCAATTTTCCGAGAGGTTTGAAGGTATAGAC   180
WX1         GGCGGTCCACCACAGACCATTCTCTTCAGGCAATTTTCCGAGAGGTTTGAAGGTATAGAC   180
WZ1         GGCGGTCCACCACAGACCATTCTCTTCAGGCAATTTTCCGAGAGGTTTGAAGGTATAGAC   180
            ****************** *****************************************

WT          TCTACTACTTCATTCTACGAAGAAGTATTCCGCTCCTCCGAGCTAGTTTCCCGACCGCAG   240
9930        TCTACTACTTCATTCTACGAAGAAGTATTCCGCTCCTCCGAGCTAGTTTCCCGACCGCAG   240
up          TCTACTACTTCATTCTACGAAGAAGTATTCCGCTCCTCCGAGCTAGTTTCCCGACCGCAG   240
B1          TCTACTACTTCATTCTACGAAGAAGTATTCCGCTCCTCCGAGCTAGTTTCCCGACCGCAG   240
CGN19839    TCTACTACTTCATTCTACGAAGAAGTATTCCGCTCCTCCGAGCTAGTTTCCCGACCGCAG   240
30          TCTACTACTTCATTCTACGAAGAAGTATTCCGCTCCTCCGAGCTAGTTTCCCGACCGCAG   240
61          TCTACTACTTCATTCTACGAAGAAGTATTCCGCTCCTCCGAGCTAGTTTCCCGACCGCAG   240
422         TCTACTACTTCATTCTACGAAGAAGTATTCCGCTCCTCCGAGCTAGTTTCCCGACCGCAG   240
L02         TCTACTACTTCATTCTACGAAGAAGTATTCCGCTCCTCCGAGCTAGTTTCCCGACCGCAG   240
L03         TCTACTACTTCATTCTACGAAGAAGTATTCCGCTCCTCCGAGCTAGTTTCCCGACCGCAG   240
S1003       TCTACTACTTCATTCTACGAAGAAGTATTCCGCTCCTCCGAGCTAGTTTCCCGACCGCAG   240
S05         TCTACTACTTCATTCTACGAAGAAGTATTCCGCTCCTCCGAGCTAGTTTCCCGACCGCAG   240
S06         TCTACTACTTCATTCTACGAAGAAGTATTCCGCTCCTCCGAGCTAGTTTCCCGACCGCAG   240
WX2         TCTACTACTTCATTCTACGAAGAAGTATTCCGCTCCTCCGAGCTAGTTTCCCGACCGCAG   240
S52         TCTACTACTTCATTCTACGAAGAAGTATTCCGCTCCTCCGAGCTAGTTTCCCGACCGCAG   240
S94         TCTACTACTTCATTCTACGAAGAAGTATTCCGCTCCTCCGAGCTAGTTTCCCGACCGCAG   240
SB-2        TCTACTACTTCATTCTACGAAGAAGTATTCCGCTCCTCCGAGCTAGTTTCCCGACCGCAG   240
SD4         TCTACTACTTCATTCTACGAAGAAGTATTCCGCTCCTCCGAGCTAGTTTCCCGACCGCAG   240
WD1         TCTACTACTTCATTCTACGAAGAAGTATTCCGCTCCTCCGAGCTAGTTTCCCGACCGCAG   240
WD2         TCTACTACTTCATTCTACGAAGAAGTATTCCGCTCCTCCGAGCTAGTTTCCCGACCGCAG   240
WX1         TCTACTACTTCATTCTACGAAGAAGTATTCCGCTCCTCCGAGCTAGTTTCCCGACCGCAG   240
WZ1         TCTACTACTTCATTCTACGAAGAAGTATTCCGCTCCTCCGAGCTAGTTTCCCGACCGCAG   240
            ***********************************************************
```

WT	AAGGGTGGCCGGAGCTTGCCTGCCTTTAGAATCCCTGTTAAGGAGGATAGATTTTACCGC	300
9930	AAGGGTGGCCGGAGCTTGCCTGCCTTTAGAATCCCTGTTAAGGAGGATAGATTTTACCGC	300
up	AAGGGTGGCCGGAGCTTGCCTGCCTTTAGAATCCCTGTTAAGGAGGATAGATTTTACCGC	300
B1	AAGGGTGGCCGGAGCTTGCCTGCCTTTAGAATCCCTGTTAAGGAGGATAGATTTTACCGC	300
CGN19839	AAGGGTGGCCGGAGCTTGCCTGCCTTTAGAATCCCTGTTAAGGAGGATAGATTTTACCGC	300
30	AAGGGTGGCCGGAGCTTGCCTGCCTTTAGAATCCCTGTTAAGGAGGATAGATTTTACCGC	300
61	AAGGGTGGCCGGAGCTTGCCTGCCTTTAGAATCCCTGTTAAGGAGGATAGATTTTACCGC	300
422	AAGGGTGGCCGGAGCTTGCCTGCCTTTAGAATCCCTGTTAAGGAGGATAGATTTTACCGC	300
L02	AAGGGTGGCCGGAGCTTGCCTGCCTTTAGAATCCCTGTTAAGGAGGATAGATTTTACCGC	300
L03	AAGGGTGGCCGGAGCTTGCCTGCCTTTAGAATCCCTGTTAAGGAGGATAGATTTTACCGC	300
S1003	AAGGGTGGCCGGAGCTTGCCTGCCTTTAGAATCCCTGTTAAGGAGGATAGATTTTACCGC	300
S05	AAGGGTGGCCGGAGCTTGCCTGCCTTTAGAATCCCTGTTAAGGAGGATAGATTTTACCGC	300
S06	AAGGGTGGCCGGAGCTTGCCTGCCTTTAGAATCCCTGTTAAGGAGGATAGATTTTACCGC	300
WX2	AAGGGTGGCCGGAGCTTGCCTGCCTTTAGAATCCCTGTTAAGGAGGATAGATTTTACCGC	300
S52	AAGGGTGGCCGGAGCTTGCCTGCCTTTAGAATCCCTGTTAAGGAGGATAGATTTTACCGC	300
S94	AAGGGTGGCCGGAGCTTGCCTGCCTTTAGAATCCCTGTTAAGGAGGATAGATTTTACCGC	300
SB-2	AAGGGTGGCCGGAGCTTGCCTGCCTTTAGAATCCCTGTTAAGGAGGATAGATTTTACCGC	300
SD4	AAGGGTGGCCGGAGCTTGCCTGCCTTTAGAATCCCTGTTAAGGAGGATAGATTTTACCGC	300
WD1	AAGGGTGGCCGGAGCTTGCCTGCCTTTAGAATCCCTGTTAAGGAGGATAGATTTTACCGC	300
WD2	AAGGGTGGCCGGAGCTTGCCTGCCTTTAGAATCCCTGTTAAGGAGGATAGATTTTACCGC	300
WX1	AAGGGTGGCCGGAGCTTGCCTGCCTTTAGAATCCCTGTTAAGGAGGATAGATTTTACCGC	300
WZ1	AAGGGTGGCCGGAGCTTGCCTGCCTTTAGAATCCCTGTTAAGGAGGATAGATTTTACCGC	300
	**	

WT	GATGTTTTTGGATCGGAAGATGGTCGACGGTCGAGAGATAGGTCGGAGCCGAGCTCTAAG	360
9930	GATGTTTTTGGATCGGAAGATGGTCGACGGTCGAGAGATAGGTCGGAGCCGAGCTCTAAG	360
up	GATGTTTTTGGATCGGAAGATGGTCGACGGTCGAGAGATAGGTCGGAGCCGAGCTCTAAG	360
B1	GATGTTTTTGGATCGGAAGATGGTCGACGGTCGAGAGATAGGTCGGAGCCGAGCTCTAAG	360
CGN19839	GATGTTTTTGGATCGGAAGATGGTCGACGGTCGAGAGATAGGTCGGAGCCGAGCTCTAAG	360
30	GATGTTTTTGGATCGGAAGATGGTCGACGGTCGAGAGATAGGTCGGAGCCGAGCTCTAAG	360
61	GATGTTTTTGGATCGGAAGATGGTCGACGGTCGAGAGATAGGTCGGAGCCGAGCTCTAAG	360
422	GATGTTTTTGGATCGGAAGATGGTCGACGGTCGAGAGATAGGTCGGAGCCGAGCTCTAAG	360
L02	GATGTTTTTGGATCGGAAGATGGTCGACGGTCGAGAGATAGGTCGGAGCCGAGCTCTAAG	360
L03	GATGTTTTTGGATCGGAAGATGGTCGACGGTCGAGAGATAGGTCGGAGCCGAGCTCTAAG	360
S1003	GATGTTTTTGGATCGGAAGATGGTCGACGGTCGAGAGATAGGTCGGAGCCGAGCTCTAAG	360
S05	GATGTTTTTGGATCGGAAGATGGTCGACGGTCGAGAGATAGGTCGGAGCCGAGCTCTAAG	360
S06	GATGTTTTTGGATCGGAAGATGGTCGACGGTCGAGAGATAGGTCGGAGCCGAGCTCTAAG	360
WX2	GATGTTTTTGGATCGGAAGATGGTCGACGGTCGAGAGATAGGTCGGAGCCGAGCTCTAAG	360
S52	GATGTTTTTGGATCGGAAGATGGTCGACGGTCGAGAGATAGGTCGGAGCCGAGCTCTAAG	360
S94	GATGTTTTTGGATCGGAAGATGGTCGACGGTCGAGAGATAGGTCGGAGCCGAGCTCTAAG	360
SB-2	GATGTTTTTGGATCGGAAGATGGTCGACGGTCGAGAGATAGGTCGGAGCCGAGCTCTAAG	360
SD4	GATGTTTTTGGATCGGAAGATGGTCGACGGTCGAGAGATAGGTCGGAGCCGAGCTCTAAG	360
WD1	GATGTTTTTGGATCGGAAGATGGTCGACGGTCGAGAGATAGGTCGGAGCCGAGCTCTAAG	360
WD2	GATGTTTTTGGATCGGAAGATGGTCGACGGTCGAGAGATAGGTCGGAGCCGAGCTCTAAG	360
WX1	GATGTTTTTGGATCGGAAGATGGTCGACGGTCGAGAGATAGGTCGGAGCCGAGCTCTAAG	360
WZ1	GATGTTTTTGGATCGGAAGATGGTCGACGGTCGAGAGATAGGTCGGAGCCGAGCTCTAAG	360
	**	

WT	GAATTCACTAGATCGAACTCGTCTTCTGATTTCACCCGTCTTCGGCCGGTCATCGGAGAT	420
9930	GAATTCACTAGATCGAACTCGTCTTCTGATTTCACCCGTCTTCGGCCGGTCATCGGAGAT	420
up	GAATTCACTAGATCGAACTCGTCTTCTGATTTCACCCGTCTTCGGCCGGTCATCGGAGAT	420
B1	GAATTCACTAGATCGAACTCGTCTTCTGATTTCACCCGTCTTCGGCCGGTCATCGGAGAT	420
CGN19839	GAATTCACTAGATCGAACTCGTCTTCTGATTTCACCCGTCTTCGGCCGGTCATCGGAGAT	420
30	GAATTCACTAGATCGAACTCGTCTTCTGATTTCACCCGTCTTCGGCCGGTCATCGGAGAT	420
61	GAATTCACTAGATCGAACTCGTCTTCTGATTTCACCCGTCTTCGGCCGGTCATCGGAGAT	420
422	GAATTCACTAGATCGAACTCGTCTTCTGATTTCACCCGTCTTCGGCCGGTCATCGGAGAT	420
L02	GAATTCACTAGATCGAACTCGTCTTCTGATTTCACCCGTCTTCGGCCGGTCATCGGAGAT	420
L03	GAATTCACTAGATCGAACTCGTCTTCTGATTTCACCCGTCTTCGGCCGGTCATCGGAGAT	420
S1003	GAATTCACTAGATCGAACTCGTCTTCTGATTTCACCCGTCTTCGGCCGGTCATCGGAGAT	420
S05	GAATTCACTAGATCGAACTCGTCTTCTGATTTCACCCGTCTTCGGCCGGTCATCGGAGAT	420
S06	GAATTCACTAGATCGAACTCGTCTTCTGATTTCACCCGTCTTCGGCCGGTCATCGGAGAT	420
WX2	GAATTCACTAGATCGAACTCGTCTTCTGATTTCACCCGTCTTCGGCCGGTCATCGGAGAT	420
S52	GAATTCACTAGATCGAACTCGTCTTCTGATTTCACCCGTCTTCGGCCGGTCATCGGAGAT	420
S94	GAATTCACTAGATCGAACTCGTCTTCTGATTTCACCCGTCTTCGGCCGGTCATCGGAGAT	420
SB-2	GAATTCACTAGATCGAACTCGTCTTCTGATTTCACCCGTCTTCGGCCGGTCATCGGAGAT	420
SD4	GAATTCACTAGATCGAACTCGTCTTCTGATTTCACCCGTCTTCGGCCGGTCATCGGAGAT	420
WD1	GAATTCACTAGATCGAACTCGTCTTCTGATTTCACCCGTCTTCGGCCGGTCATCGGAGAT	420
WD2	GAATTCACTAGATCGAACTCGTCTTCTGATTTCACCCGTCTTCGGCCGGTCATCGGAGAT	420
WX1	GAATTCACTAGATCGAACTCGTCTTCTGATTTCACCCGTCTTCGGCCGGTCATCGGAGAT	420
WZ1	GAATTCACTAGATCGAACTCGTCTTCTGATTTCACCCGTCTTCGGCCGGTCATCGGAGAT	420
	**	

WT	GACGTGGCGTTCCCTTCGTCGTCTTCAAATCACAGGCCAACCAATGTCCCAACACAATGG	480
9930	GACGTGGCGTTCCCTTCGTCGTCTTCAAATCACAGGCCAACCAATGTCCCAACACAATGG	480
up	GACGTGGCGTTCCCTTCGTCGTCTTCAAATCACAGGCCAACCAATGTCCCAACACAATGG	480
B1	GACGTGGCGTTCCCTTCGTCGTCTTCAAATCACAGGCCAACCAATGTCCCAACACAATGG	480
CGN19839	GACGTGGCGTTCCCTTCGTCGTCTTCAAATCACAGGCCAACCAATGTCCCAACACAATGG	480
30	GACGTGGCGTTCCCTTCGTCGTCTTCAAATCACAGGCCAACCAATGTCCCAACACAATGG	480
61	GACGTGGCGTTCCCTTCGTCGTCTTCAAATCACAGGCCAACCAATGTCCCAACACAATGG	480
422	GACGTGGCGTTCCCTTCGTCGTCTTCAAATCACAGGCCAACCAATGTCCCAACACAATGG	480
L02	GACGTGGCGTTCCCTTCGTCGTCTTCAAATCACAGGCCAACCAATGTCCCAACACAATGG	480
L03	GACGTGGCGTTCCCTTCGTCGTCTTCAAATCACAGGCCAACCAATGTCCCAACACAATGG	480
S1003	GACGTGGCGTTCCCTTCGTCGTCTTCAAATCACAGGCCAACCAATGTCCCAACACAATGG	480
S05	GACGTGGCGTTCCCTTCGTCGTCTTCAAATCACAGGCCAACCAATGTCCCAACACAATGG	480
S06	GACGTGGCGTTCCCTTCGTCGTCTTCAAATCACAGGCCAACCAATGTCCCAACACAATGG	480
WX2	GACGTGGCGTTCCCTTCGTCGTCTTCAAATCACAGGCCAACCAATGTCCCAACACAATGG	480
S52	GACGTGGCGTTCCCTTCGTCGTCTTCAAATCACAGGCCAACCAATGTCCCAACACAATGG	480
S94	GACGTGGCGTTCCCTTCGTCGTCTTCAAATCACAGGCCAACCAATGTCCCAACACAATGG	480
SB-2	GACGTGGCGTTCCCTTCGTCGTCTTCAAATCACAGGCCAACCAATGTCCCAACACAATGG	480
SD4	GACGTGGCGTTCCCTTCGTCGTCTTCAAATCACAGGCCAACCAATGTCCCAACACAATGG	480
WD1	GACGTGGCGTTCCCTTCGTCGTCTTCAAATCACAGGCCAACCAATGTCCCAACACAATGG	480
WD2	GACGTGGCGTTCCCTTCGTCGTCTTCAAATCACAGGCCAACCAATGTCCCAACACAATGG	480
WX1	GACGTGGCGTTCCCTTCGTCGTCTTCAAATCACAGGCCAACCAATGTCCCAACACAATGG	480
WZ1	GACGTGGCGTTCCCTTCGTCGTCTTCAAATCACAGGCCAACCAATGTCCCAACACAATGG	480
	**	

```
WT         AATTCATACACAACTATGTTCAAGGAACAGGAAATGCCACAGTTTGCACCTCATCTCTCC   540
9930       AATTCATACACAACTATGTTCAAGGAACAGGAAATGCCACAGTTTGCACCTCATCTCTCC   540
up         AATTCATACA-----ATGTTCAAGGAACAGGAAATGCCACAGTTTGCACCTCATCTCTCC   535
B1         AATTCATACA-----ATGTTCAAGGAACAGGAAATGCCACAGTTTGCACCTCATCTCTCC   535
CGN19839   AATTCATACACAACTATGTTCAAGGAACAGGAAATGCCACAGTTTGCACCTCATCTCTCC   540
30         AATTCATACACAACTATGTTCAAGGAACAGGAAATGCCACAGTTTGCACCTCATCTCTCC   540
61         AATTCATACACAACTATGTTCAAGGAACAGGAAATGCCACAGTTTGCACCTCATCTCTCC   540
422        AATTCATACACAACTATGTTCAAGGAACAGGAAATGCCACAGTTTGCACCTCATCTCTCC   540
L02        AATTCATACACAACTATGTTCAAGGAACAGGAAATGCCACAGTTTGCACCTCATCTCTCC   540
L03        AATTCATACACAACTATGTTCAAGGAACAGGAAATGCCACAGTTTGCACCTCATCTCTCC   540
S1003      AATTCATACACAACTATGTTCAAGGAACAGGAAATGCCACAGTTTGCACCTCATCTCTCC   540
S05        AATTCATACACAACTATGTTCAAGGAACAGGAAATGCCACAGTTTGCACCTCATCTCTCC   540
S06        AATTCATACACAACTATGTTCAAGGAACAGGAAATGCCACAGTTTGCACCTCATCTCTCC   540
WX2        AATTCATACACAACTATGTTCAAGGAACAGGAAATGCCACAGTTTGCACCTCATCTCTCC   540
S52        AATTCATACACAACTATGTTCAAGGAACAGGAAATGCCACAGTTTGCACCTCATCTCTCC   540
S94        AATTCATACACAACTATGTTCAAGGAACAGGAAATGCCACAGTTTGCACCTCATCTCTCC   540
SB-2       AATTCATACACAACTATGTTCAAGGAACAGGAAATGCCACAGTTTGCACCTCATCTCTCC   540
SD4        AATTCATACACAACTATGTTCAAGGAACAGGAAATGCCACAGTTTGCACCTCATCTCTCC   540
WD1        AATTCATACACAACTATGTTCAAGGAACAGGAAATGCCACAGTTTGCACCTCATCTCTCC   540
WD2        AATTCATACACAACTATGTTCAAGGAACAGGAAATGCCACAGTTTGCACCTCATCTCTCC   540
WX1        AATTCATACACAACTATGTTCAAGGAACAGGAAATGCCACAGTTTGCACCTCATCTCTCC   540
WZ1        AATTCATACACAACTATGTTCAAGGAACAGGAAATGCCACAGTTTGCACCTCATCTCTCC   540
           *********      *********************************************

WT         CCTCATATGGATAACCGTTATGTAGAAGATGAATATGATGATAGATACAAAAGCTCAGAC   600
9930       CCTCATATGGATAACCGTTATGTAGAAGATGAATATGATGATAGATACAAAAGCTCAGAC   600
up         CCTCATATGGATAACCGTTATGTAGAAGATGAATATGATGATAGATACAAAAGCTCAGAC   595
B1         CCTCATATGGATAACCGTTATGTAGAAGATGAATATGATGATAGATACAAAAGCTCAGAC   595
CGN19839   CCTCATATGGATAACCGTTATGTAGAAGATGAATATGATGATAGATACAAAAGCTCAGAC   600
30         CCTCATATGGATAACCGTTATGTAGAAGATGAATATGATGATAGATACAAAAGCTCAGAC   600
61         CCTCATATGGATAACCGTTATGTAGAAGATGAATATGATGATAGATACAAAAGCTCAGAC   600
422        CCTCATATGGATAACCGTTATGTAGAAGATGAATATGATGATAGATACAAAAGCTCAGAC   600
L02        CCTCATATGGATAACCGTTATGTAGAAGATGAATATGATGATAGATACAAAAGCTCAGAC   600
L03        CCTCATATGGATAACCGTTATGTAGAAGATGAATATGATGATAGATACAAAAGCTCAGAC   600
S1003      CCTCATATGGATAACCGTTATGTAGAAGATGAATATGATGATAGATACAAAAGCTCAGAC   600
S05        CCTCATATGGATAACCGTTATGTAGAAGATGAATATGATGATAGATACAAAAGCTCAGAC   600
S06        CCTCATATGGATAACCGTTATGTAGAAGATGAATATGATGATAGATACAAAAGCTCAGAC   600
WX2        CCTCATATGGATAACCGTTATGTAGAAGATGAATATGATGATAGATACAAAAGCTCAGAC   600
S52        CCTCATATGGATAACCGTTATGTAGAAGATGAATATGATGATAGATACAAAAGCTCAGAC   600
S94        CCTCATATGGATAACCGTTATGTAGAAGATGAATATGATGATAGATACAAAAGCTCAGAC   600
SB-2       CCTCATATGGATAACCGTTATGTAGAAGATGAATATGATGATAGATACAAAAGCTCAGAC   600
SD4        CCTCATATGGATAACCGTTATGTAGAAGATGAATATGATGATAGATACAAAAGCTCAGAC   600
WD1        CCTCATATGGATAACCGTTATGTAGAAGATGAATATGATGATAGATACAAAAGCTCAGAC   600
WD2        CCTCATATGGATAACCGTTATGTAGAAGATGAATATGATGATAGATACAAAAGCTCAGAC   600
WX1        CCTCATATGGATAACCGTTATGTAGAAGATGAATATGATGATAGATACAAAAGCTCAGAC   600
WZ1        CCTCATATGGATAACCGTTATGTAGAAGATGAATATGATGATAGATACAAAAGCTCAGAC   600
           ************************************************************
```

```
WT          CATGGATTTGGACAGCCTGTATCATCGCCAGAAACCGTTATTCTGGAACCAAATTCGTTC   660
9930        CATGGATTTGGACAGCCTGTATCATCGCCAGAAACCGTTATTCTGGAACCAAATTCGTTC   660
up          CATGGATTTGGACAGCCTGTATCATCGCCAGAAACCGTTATTCTGGAACCAAATTCGTTC   655
B1          CATGGATTTGGACAGCCTGTATCATCGCCAGAAACCGTTATTCTGGAACCAAATTCGTTC   655
CGN19839    CATGGATTTGGACAGCCTGTATCATCGCCAGAAACCGTTATTCTGGAACCAAATTCGTTC   660
30          CATGGATTTGGACAGCCTGTATCATCGCCAGAAACCGTTATTCTGGAACCAAATTCGTTC   660
61          CATGGATTTGGACAGCCTGTATCATCGCCAGAAACCGTTATTCTGGAACCAAATTCGTTC   660
422         CATGGATTTGGACAGCCTGTATCATCGCCAGAAACCGTTATTCTGGAACCAAATTCGTTC   660
L02         CATGGATTTGGACAGCCTGTATCATCGCCAGAAACCGTTATTCTGGAACCAAATTCGTTC   660
L03         CATGGATTTGGACAGCCTGTATCATCGCCAGAAACCGTTATTCTGGAACCAAATTCGTTC   660
S1003       CATGGATTTGGACAGCCTGTATCATCGCCAGAAACCGTTATTCTGGAACCAAATTCGTTC   660
S05         CATGGATTTGGACAGCCTGTATCATCGCCAGAAACCGTTATTCTGGAACCAAATTCGTTC   660
S06         CATGGATTTGGACAGCCTGTATCATCGCCAGAAACCGTTATTCTGGAACCAAATTCGTTC   660
WX2         CATGGATTTGGACAGCCTGTATCATCGCCAGAAACCGTTATTCTGGAACCAAATTCGTTC   660
S52         CATGGATTTGGACAGCCTGTATCATCGCCAGAAACCGTTATTCTGGAACCAAATTCGTTC   660
S94         CATGGATTTGGACAGCCTGTATCATCGCCAGAAACCGTTATTCTGGAACCAAATTCGTTC   660
SB-2        CATGGATTTGGACAGCCTGTATCATCGCCAGAAACCGTTATTCTGGAACCAAATTCGTTC   660
SD4         CATGGATTTGGACAGCCTGTATCATCGCCAGAAACCGTTATTCTGGAACCAAATTCGTTC   660
WD1         CATGGATTTGGACAGCCTGTATCATCGCCAGAAACCGTTATTCTGGAACCAAATTCGTTC   660
WD2         CATGGATTTGGACAGCCTGTATCATCGCCAGAAACCGTTATTCTGGAACCAAATTCGTTC   660
WX1         CATGGATTTGGACAGCCTGTATCATCGCCAGAAACCGTTATTCTGGAACCAAATTCGTTC   660
WZ1         CATGGATTTGGACAGCCTGTATCATCGCCAGAAACCGTTATTCTGGAACCAAATTCGTTC   660
            ************************************************************

WT          AGAAGCATCAAGATCTGCGTGGATGATTATTTAGAAATAAACTCCCCATCATCTCCTGAA   720
9930        AGAAGCATCAAGATCTGCGTGGATGATTATTTAGAAATAAACTCCCCATCATCTCCTGAA   720
up          AGAAGCATCAAGATCTGCGTGGATGATTATTTAGAAATAAACTCCCCATCATCTCCTGAA   715
B1          AGAAGCATCAAGATCTGCGTGGATGATTATTTAGAAATAAACTCCCCATCATCTCCTGAA   715
CGN19839    AGAAGCATCAAGATCTGCGTGGATGATTATTTAGAAATAAACTCCCCATCATCTCCTGAA   720
30          AGAAGCATCAAGATCTGCGTGGATGATTATTTAGAAATAAACTCCCCATCATCTCCTGAA   720
61          AGAAGCATCAAGATCTGCGTGGATGATTATTTAGAAATAAACTCCCCATCATCTCCTGAA   720
422         AGAAGCATCAAGATCTGCGTGGATGATTATTTAGAAATAAACTCCCCATCATCTCCTGAA   720
L02         AGAAGCATCAAGATCTGCGTGGATGATTATTTAGAAATAAACTCCCCATCATCTCCTGAA   720
L03         AGAAGCATCAAGATCTGCGTGGATGATTATTTAGAAATAAACTCCCCATCATCTCCTGAA   720
S1003       AGAAGCATCAAGATCTGCGTGGATGATTATTTAGAAATAAACTCCCCATCATCTCCTGAA   720
S05         AGAAGCATCAAGATCTGCGTGGATGATTATTTAGAAATAAACTCCCCATCATCTCCTGAA   720
S06         AGAAGCATCAAGATCTGCGTGGATGATTATTTAGAAATAAACTCCCCATCATCTCCTGAA   720
WX2         AGAAGCATCAAGATCTGCGTGGATGATTATTTAGAAATAAACTCCCCATCATCTCCTGAA   720
S52         AGAAGCATCAAGATCTGCGTGGATGATTATTTAGAAATAAACTCCCCATCATCTCCTGAA   720
S94         AGAAGCATCAAGATCTGCGTGGATGATTATTTAGAAATAAACTCCCCATCATCTCCTGAA   720
SB-2        AGAAGCATCAAGATCTGCGTGGATGATTATTTAGAAATAAACTCCCCATCATCTCCTGAA   720
SD4         AGAAGCATCAAGATCTGCGTGGATGATTATTTAGAAATAAACTCCCCATCATCTCCTGAA   720
WD1         AGAAGCATCAAGATCTGCGTGGATGATTATTTAGAAATAAACTCCCCATCATCTCCTGAA   720
WD2         AGAAGCATCAAGATCTGCGTGGATGATTATTTAGAAATAAACTCCCCATCATCTCCTGAA   720
WX1         AGAAGCATCAAGATCTGCGTGGATGATTATTTAGAAATAAACTCCCCATCATCTCCTGAA   720
WZ1         AGAAGCATCAAGATCTGCGTGGATGATTATTTAGAAATAAACTCCCCATCATCTCCTGAA   720
            ************************************************************
```

WT	TCTTCTCTCTGTGAGGATCCAGTTTATTATGATGGAACTTACTGTAATGTTTTACCGGAA	780
9930	TCTTCTCTCTGTGAGGATCCAGTTTATTATGATGGAACTTACTGTAATGTTTTACCGGAA	780
up	TCTTCTCTCTGTGAGGATCCAGTTTATTATGATGGAACTTACTGTAATGTTTTACCGGAA	775
B1	TCTTCTCTCTGTGAGGATCCAGTTTATTATGATGGAACTTACTGTAATGTTTTACCGGAA	775
CGN19839	TCTTCTCTCTGTGAGGATCCAGTTTATTATGATGGAACTTACTGTAATGTTTTACCGGAA	780
30	TCTTCTCTCTGTGAGGATCCAGTTTATTATGATGGAACTTACTGTAATGTTTTACCGGAA	780
61	TCTTCTCTCTGTGAGGATCCAGTTTATTATGATGGAACTTACTGTAATGTTTTACCGGAA	780
422	TCTTCTCTCTGTGAGGATCCAGTTTATTATGATGGAACTTACTGTAATGTTTTACCGGAA	780
L02	TCTTCTCTCTGTGAGGATCCAGTTTATTATGATGGAACTTACTGTAATGTTTTACCGGAA	780
L03	TCTTCTCTCTGTGAGGATCCAGTTTATTATGATGGAACTTACTGTAATGTTTTACCGGAA	780
S1003	TCTTCTCTCTGTGAGGATCCAGTTTATTATGATGGAACTTACTGTAATGTTTTACCGGAA	780
S05	TCTTCTCTCTGTGAGGATCCAGTTTATTATGATGGAACTTACTGTAATGTTTTACCGGAA	780
S06	TCTTCTCTCTGTGAGGATCCAGTTTATTATGATGGAACTTACTGTAATGTTTTACCGGAA	780
WX2	TCTTCTCTCTGTGAGGATCCAGTTTATTATGATGGAACTTACTGTAATGTTTTACCGGAA	780
S52	TCTTCTCTCTGTGAGGATCCAGTTTATTATGATGGAACTTACTGTAATGTTTTACCGGAA	780
S94	TCTTCTCTCTGTGAGGATCCAGTTTATTATGATGGAACTTACTGTAATGTTTTACCGGAA	780
SB-2	TCTTCTCTCTGTGAGGATCCAGTTTATTATGATGGAACTTACTGTAATGTTTTACCGGAA	780
SD4	TCTTCTCTCTGTGAGGATCCAGTTTATTATGATGGAACTTACTGTAATGTTTTACCGGAA	780
WD1	TCTTCTCTCTGTGAGGATCCAGTTTATTATGATGGAACTTACTGTAATGTTTTACCGGAA	780
WD2	TCTTCTCTCTGTGAGGATCCAGTTTATTATGATGGAACTTACTGTAATGTTTTACCGGAA	780
WX1	TCTTCTCTCTGTGAGGATCCAGTTTATTATGATGGAACTTACTGTAATGTTTTACCGGAA	780
WZ1	TCTTCTCTCTGTGAGGATCCAGTTTATTATGATGGAACTTACTGTAATGTTTTACCGGAA	780
	**	

WT	GATGACGATGACGATGAAGATGCTATGAGCTCTTATGTCATTGAGATAACTTCTATCAAT	840
9930	GATGACGATGACGATGAAGATGCTATGAGCTCTTATGTCATTGAGATAACTTCTATCAAT	840
up	GATGACGATGACGATGAAGATGCTATGAGCTCTTATGTCATTGAGATAACTTCTATCAAT	835
B1	GATGACGATGACGATGAAGATGCTATGAGCTCTTATGTCATTGAGATAACTTCTATCAAT	835
CGN19839	GATGACGATGACGATGAAGATGCTATGAGCTCTTATGTCATTGAGATAACTTCTATCAAT	840
30	GATGACGATGACGATGAAGATGCTATGAGCTCTTATGTCATTGAGATAACTTCTATCAAT	840
61	GATGACGATGACGATGAAGATGCTATGAGCTCTTATGTCATTGAGATAACTTCTATCAAT	840
422	GATGACGATGACGATGAAGATGCTATGAGCTCTTATGTCATTGAGATAACTTCTATCAAT	840
L02	GATGACGATGACGATGAAGATGCTATGAGCTCTTATGTCATTGAGATAACTTCTATCAAT	840
L03	GATGACGATGACGATGAAGATGCTATGAGCTCTTATGTCATTGAGATAACTTCTATCAAT	840
S1003	GATGACGATGACGATGAAGATGCTATGAGCTCTTATGTCATTGAGATAACTTCTATCAAT	840
S05	GATGACGATGACGATGAAGATGCTATGAGCTCTTATGTCATTGAGATAACTTCTATCAAT	840
S06	GATGACGATGACGATGAAGATGCTATGAGCTCTTATGTCATTGAGATAACTTCTATCAAT	840
WX2	GATGACGATGACGATGAAGATGCTATGAGCTCTTATGTCATTGAGATAACTTCTATCAAT	840
S52	GATGACGATGACGATGAAGATGCTATGAGCTCTTATGTCATTGAGATAACTTCTATCAAT	840
S94	GATGACGATGACGATGAAGATGCTATGAGCTCTTATGTCATTGAGATAACTTCTATCAAT	840
SB-2	GATGACGATGACGATGAAGATGCTATGAGCTCTTATGTCATTGAGATAACTTCTATCAAT	840
SD4	GATGACGATGACGATGAAGATGCTATGAGCTCTTATGTCATTGAGATAACTTCTATCAAT	840
WD1	GATGACGATGACGATGAAGATGCTATGAGCTCTTATGTCATTGAGATAACTTCTATCAAT	840
WD2	GATGACGATGACGATGAAGATGCTATGAGCTCTTATGTCATTGAGATAACTTCTATCAAT	840
WX1	GATGACGATGACGATGAAGATGCTATGAGCTCTTATGTCATTGAGATAACTTCTATCAAT	840
WZ1	GATGACGATGACGATGAAGATGCTATGAGCTCTTATGTCATTGAGATAACTTCTATCAAT	840
	**	

WT	AGAGAAGAATATAGAGAAGAAGTTTCTATTGACGAAGCAATTGCTTGGGCTAAATCGAAG	900
9930	AGAGAAGAATATAGAGAAGAAGTTTCTATTGACGAAGCAATTGCTTGGGCTAAATCGAAG	900
up	AGAGAAGAATATAGAGAAGAAGTTTCTATTGACGAAGCAATTGCTTGGGCTAAATCGAAG	895
B1	AGAGAAGAATATAGAGAAGAAGTTTCTATTGACGAAGCAATTGCTTGGGCTAAATCGAAG	895
CGN19839	AGAGAAGAATATAGAGAAGAAGTTTCTATTGACGAAGCAATTGCTTGGGCTAAATCGAAG	900
30	AGAGAAGAATATAGAGAAGAAGTTTCTATTGACGAAGCAATTGCTTGGGCTAAATCGAAG	900
61	AGAGAAGAATATAGAGAAGAAGTTTCTATTGACGAAGCAATTGCTTGGGCTAAATCGAAG	900
422	AGAGAAGAATATAGAGAAGAAGTTTCTATTGACGAAGCAATTGCTTGGGCTAAATCGAAG	900
L02	AGAGAAGAATATAGAGAAGAAGTTTCTATTGACGAAGCAATTGCTTGGGCTAAATCGAAG	900
L03	AGAGAAGAATATAGAGAAGAAGTTTCTATTGACGAAGCAATTGCTTGGGCTAAATCGAAG	900
S1003	AGAGAAGAATATAGAGAAGAAGTTTCTATTGACGAAGCAATTGCTTGGGCTAAATCGAAG	900
S05	AGAGAAGAATATAGAGAAGAAGTTTCTATTGACGAAGCAATTGCTTGGGCTAAATCGAAG	900
S06	AGAGAAGAATATAGAGAAGAAGTTTCTATTGACGAAGCAATTGCTTGGGCTAAATCGAAG	900
WX2	AGAGAAGAATATAGAGAAGAAGTTTCTATTGACGAAGCAATTGCTTGGGCTAAATCGAAG	900
S52	AGAGAAGAATATAGAGAAGAAGTTTCTATTGACGAAGCAATTGCTTGGGCTAAATCGAAG	900
S94	AGAGAAGAATATAGAGAAGAAGTTTCTATTGACGAAGCAATTGCTTGGGCTAAATCGAAG	900
SB-2	AGAGAAGAATATAGAGAAGAAGTTTCTATTGACGAAGCAATTGCTTGGGCTAAATCGAAG	900
SD4	AGAGAAGAATATAGAGAAGAAGTTTCTATTGACGAAGCAATTGCTTGGGCTAAATCGAAG	900
WD1	AGAGAAGAATATAGAGAAGAAGTTTCTATTGACGAAGCAATTGCTTGGGCTAAATCGAAG	900
WD2	AGAGAAGAATATAGAGAAGAAGTTTCTATTGACGAAGCAATTGCTTGGGCTAAATCGAAG	900
WX1	AGAGAAGAATATAGAGAAGAAGTTTCTATTGACGAAGCAATTGCTTGGGCTAAATCGAAG	900
WZ1	AGAGAAGAATATAGAGAAGAAGTTTCTATTGACGAAGCAATTGCTTGGGCTAAATCGAAG	900
	**	

WT	TATCAAAGTGCATCTGAGACAGATTTGAGTGTTAGACAACAAGAAAGCGAGCAATCTGGG	960
9930	TATCAAAGTGCATCTGAGACAGATTTGAGTGTTAGACAACAAGAAAGCGAGCAATCTGGG	960
up	TATCAAAGTGCATCTGAGACAGATTTGAGTGTTAGACAACAAGAAAGCGAGCAATCTGGG	955
B1	TATCAAAGTGCATCTGAGACAGATTTGAGTGTTAGACAACAAGAAAGCGAGCAATCTGGG	955
CGN19839	TATCAAAGTGCATCTGAGACAGATTTGAGTGTTAGACAACAAGAAAGCGAGCAATCTGGG	960
30	TATCAAAGTGCATCTGAGACAGATTTGAGTGTTAGACAACAAGAAAGCGAGCAATCTGGG	960
61	TATCAAAGTGCATCTGAGACAGATTTGAGTGTTAGACAACAAGAAAGCGAGCAATCTGGG	960
422	TATCAAAGTGCATCTGAGACAGATTTGAGTGTTAGACAACAAGAAAGCGAGCAATCTGGG	960
L02	TATCAAAGTGCATCTGAGACAGATTTGAGTGTTAGACAACAAGAAAGCGAGCAATCTGGG	960
L03	TATCAAAGTGCATCTGAGACAGATTTGAGTGTTAGACAACAAGAAAGCGAGCAATCTGGG	960
S1003	TATCAAAGTGCATCTGAGACAGATTTGAGTGTTAGACAACAAGAAAGCGAGCAATCTGGG	960
S05	TATCAAAGTGCATCTGAGACAGATTTGAGTGTTAGACAACAAGAAAGCGAGCAATCTGGG	960
S06	TATCAAAGTGCATCTGAGACAGATTTGAGTGTTAGACAACAAGAAAGCGAGCAATCTGGG	960
WX2	TATCAAAGTGCATCTGAGACAGATTTGAGTGTTAGACAACAAGAAAGCGAGCAATCTGGG	960
S52	TATCAAAGTGCATCTGAGACAGATTTGAGTGTTAGACAACAAGAAAGCGAGCAATCTGGG	960
S94	TATCAAAGTGCATCTGAGACAGATTTGAGTGTTAGACAACAAGAAAGCGAGCAATCTGGG	960
SB-2	TATCAAAGTGCATCTGAGACAGATTTGAGTGTTAGACAACAAGAAAGCGAGCAATCTGGG	960
SD4	TATCAAAGTGCATCTGAGACAGATTTGAGTGTTAGACAACAAGAAAGCGAGCAATCTGGG	960
WD1	TATCAAAGTGCATCTGAGACAGATTTGAGTGTTAGACAACAAGAAAGCGAGCAATCTGGG	960
WD2	TATCAAAGTGCATCTGAGACAGATTTGAGTGTTAGACAACAAGAAAGCGAGCAATCTGGG	960
WX1	TATCAAAGTGCATCTGAGACAGATTTGAGTGTTAGACAACAAGAAAGCGAGCAATCTGGG	960
WZ1	TATCAAAGTGCATCTGAGACAGATTTGAGTGTTAGACAACAAGAAAGCGAGCAATCTGGG	960
	**	

WT	GAAGAAGAAGGAAGACCTGTTGCATTTGAATGCTCAGATCAGCAGTCGAATGGAAATGGA	1020
9930	GAAGAAGAAGGAAGACCTGTTGCATTTGAATGCTCAGATCAGCAGTCGAATGGAAATGGA	1020
up	GAAGAAGAAGGAAGACCTGTTGCATTTGAATGCTCAGATCAGCAGTCGAATGGAAATGGA	1015
B1	GAAGAAGAAGGAAGACCTGTTGCATTTGAATGCTCAGATCAGCAGTCGAATGGAAATGGA	1015
CGN19839	GAAGAAGAAGGAAGACCTGTTGCATTTGAATGCTCAGATCAGCAGTCGAATGGAAATGGA	1020
30	GAAGAAGAAGGAAGACCTGTTGCATTTGAATGCTCAGATCAGCAGTCGAATGGAAATGGA	1020
61	GAAGAAGAAGGAAGACCTGTTGCATTTGAATGCTCAGATCAGCAGTCGAATGGAAATGGA	1020
422	GAAGAAGAAGGAAGACCTGTTGCATTTGAATGCTCAGATCAGCAGTCGAATGGAAATGGA	1020
L02	GAAGAAGAAGGAAGACCTGTTGCATTTGAATGCTCAGATCAGCAGTCGAATGGAAATGGA	1020
L03	GAAGAAGAAGGAAGACCTGTTGCATTTGAATGCTCAGATCAGCAGTCGAATGGAAATGGA	1020
S1003	GAAGAAGAAGGAAGACCTGTTGCATTTGAATGCTCAGATCAGCAGTCGAATGGAAATGGA	1020
S05	GAAGAAGAAGGAAGACCTGTTGCATTTGAATGCTCAGATCAGCAGTCGAATGGAAATGGA	1020
S06	GAAGAAGAAGGAAGACCTGTTGCATTTGAATGCTCAGATCAGCAGTCGAATGGAAATGGA	1020
WX2	GAAGAAGAAGGAAGACCTGTTGCATTTGAATGCTCAGATCAGCAGTCGAATGGAAATGGA	1020
S52	GAAGAAGAAGGAAGACCTGTTGCATTTGAATGCTCAGATCAGCAGTCGAATGGAAATGGA	1020
S94	GAAGAAGAAGGAAGACCTGTTGCATTTGAATGCTCAGATCAGCAGTCGAATGGAAATGGA	1020
SB-2	GAAGAAGAAGGAAGACCTGTTGCATTTGAATGCTCAGATCAGCAGTCGAATGGAAATGGA	1020
SD4	GAAGAAGAAGGAAGACCTGTTGCATTTGAATGCTCAGATCAGCAGTCGAATGGAAATGGA	1020
WD1	GAAGAAGAAGGAAGACCTGTTGCATTTGAATGCTCAGATCAGCAGTCGAATGGAAATGGA	1020
WD2	GAAGAAGAAGGAAGACCTGTTGCATTTGAATGCTCAGATCAGCAGTCGAATGGAAATGGA	1020
WX1	GAAGAAGAAGGAAGACCTGTTGCATTTGAATGCTCAGATCAGCAGTCGAATGGAAATGGA	1020
WZ1	GAAGAAGAAGGAAGACCTGTTGCATTTGAATGCTCAGATCAGCAGTCGAATGGAAATGGA	1020
	**	

WT	TTGTCGCAGACTGCAGAGACACAACAGAGAGAAGTAAAAGTTGAAGAAGAAAAGCCACAG	1080
9930	TTGTCGCAGACTGCAGAGACACAACAGAGAGAAGTAAAAGTTGAAGAAGAAAAGCCACAG	1080
up	TTGTCGCAGACTGCAGAGACACAACAGAGAGAAGTAAAAGTTGAAGAAGAAAAGCCACAG	1075
B1	TTGTCGCAGACTGCAGAGACACAACAGAGAGAAGTAAAAGTTGAAGAAGAAAAGCCACAG	1075
CGN19839	TTGTCGCAGACTGCAGAGACACAACAGAGAGAAGTAAAAGTTGAAGAAGAAAAGCCACAG	1080
30	TTGTCGCAGACTGCAGAGACACAACAGAGAGAAGTAAAAGTTGAAGAAGAAAAGCCACAG	1080
61	TTGTCGCAGACTGCAGAGACACAACAGAGAGAAGTAAAAGTTGAAGAAGAAAAGCCACAG	1080
422	TTGTCGCAGACTGCAGAGACACAACAGAGAGAAGTAAAAGTTGAAGAAGAAAAGCCACAG	1080
L02	TTGTCGCAGACTGCAGAGACACAACAGAGAGAAGTAAAAGTTGAAGAAGAAAAGCCACAG	1080
L03	TTGTCGCAGACTGCAGAGACACAACAGAGAGAAGTAAAAGTTGAAGAAGAAAAGCCACAG	1080
S1003	TTGTCGCAGACTGCAGAGACACAACAGAGAGAAGTAAAAGTTGAAGAAGAAAAGCCACAG	1080
S05	TTGTCGCAGACTGCAGAGACACAACAGAGAGAAGTAAAAGTTGAAGAAGAAAAGCCACAG	1080
S06	TTGTCGCAGACTGCAGAGACACAACAGAGAGAAGTAAAAGTTGAAGAAGAAAAGCCACAG	1080
WX2	TTGTCGCAGACTGCAGAGACACAACAGAGAGAAGTAAAAGTTGAAGAAGAAAAGCCACAG	1080
S52	TTGTCGCAGACTGCAGAGACACAACAGAGAGAAGTAAAAGTTGAAGAAGAAAAGCCACAG	1080
S94	TTGTCGCAGACTGCAGAGACACAACAGAGAGAAGTAAAAGTTGAAGAAGAAAAGCCACAG	1080
SB-2	TTGTCGCAGACTGCAGAGACACAACAGAGAGAAGTAAAAGTTGAAGAAGAAAAGCCACAG	1080
SD4	TTGTCGCAGACTGCAGAGACACAACAGAGAGAAGTAAAAGTTGAAGAAGAAAAGCCACAG	1080
WD1	TTGTCGCAGACTGCAGAGACACAACAGAGAGAAGTAAAAGTTGAAGAAGAAAAGCCACAG	1080
WD2	TTGTCGCAGACTGCAGAGACACAACAGAGAGAAGTAAAAGTTGAAGAAGAAAAGCCACAG	1080
WX1	TTGTCGCAGACTGCAGAGACACAACAGAGAGAAGTAAAAGTTGAAGAAGAAAAGCCACAG	1080
WZ1	TTGTCGCAGACTGCAGAGACACAACAGAGAGAAGTAAAAGTTGAAGAAGAAAAGCCACAG	1080
	**	

WT	TTGAACATCGATAGAGAATTGGAAGGATTAGATGAAAAAATAAAGCTATGGTCAGCTGGC	1140
9930	TTGAACATCGATAGAGAATTGGAAGGATTAGATGAAAAAATAAAGCTATGGTCAGCTGGC	1140
up	TTGAACATCGATAGAGAATTGGAAGGATTAGATGAAAAAATAAAGCTATGGTCAGCTGGC	1135
B1	TTGAACATCGATAGAGAATTGGAAGGATTAGATGAAAAAATAAAGCTATGGTCAGCTGGC	1135
CGN19839	TTGAACATCGATAGAGAATTGGAAGGATTAGATGAAAAAATAAAGCTATGGTCAGCTGGC	1140
30	TTGAACATCGATAGAGAATTGGAAGGATTAGATGAAAAAATAAAGCTATGGTCAGCTGGC	1140
61	TTGAACATCGATAGAGAATTGGAAGGATTAGATGAAAAAATAAAGCTATGGTCAGCTGGC	1140
422	TTGAACATCGATAGAGAATTGGAAGGATTAGATGAAAAAATAAAGCTATGGTCAGCTGGC	1140
L02	TTGAACATCGATAGAGAATTGGAAGGATTAGATGAAAAAATAAAGCTATGGTCAGCTGGC	1140
L03	TTGAACATCGATAGAGAATTGGAAGGATTAGATGAAAAAATAAAGCTATGGTCAGCTGGC	1140
S1003	TTGAACATCGATAGAGAATTGGAAGGATTAGATGAAAAAATAAAGCTATGGTCAGCTGGC	1140
S05	TTGAACATCGATAGAGAATTGGAAGGATTAGATGAAAAAATAAAGCTATGGTCAGCTGGC	1140
S06	TTGAACATCGATAGAGAATTGGAAGGATTAGATGAAAAAATAAAGCTATGGTCAGCTGGC	1140
WX2	TTGAACATCGATAGAGAATTGGAAGGATTAGATGAAAAAATAAAGCTATGGTCAGCTGGC	1140
S52	TTGAACATCGATAGAGAATTGGAAGGATTAGATGAAAAAATAAAGCTATGGTCAGCTGGC	1140
S94	TTGAACATCGATAGAGAATTGGAAGGATTAGATGAAAAAATAAAGCTATGGTCAGCTGGC	1140
SB-2	TTGAACATCGATAGAGAATTGGAAGGATTAGATGAAAAAATAAAGCTATGGTCAGCTGGC	1140
SD4	TTGAACATCGATAGAGAATTGGAAGGATTAGATGAAAAAATAAAGCTATGGTCAGCTGGC	1140
WD1	TTGAACATCGATAGAGAATTGGAAGGATTAGATGAAAAAATAAAGCTATGGTCAGCTGGC	1140
WD2	TTGAACATCGATAGAGAATTGGAAGGATTAGATGAAAAAATAAAGCTATGGTCAGCTGGC	1140
WX1	TTGAACATCGATAGAGAATTGGAAGGATTAGATGAAAAAATAAAGCTATGGTCAGCTGGC	1140
WZ1	TTGAACATCGATAGAGAATTGGAAGGATTAGATGAAAAAATAAAGCTATGGTCAGCTGGC	1140
	**	

WT	AAGGAGACCAACATCCGCTTGCTACTTTCTACACTTCATTATATATTGTGGTCAAGTAGT	1200
9930	AAGGAGACCAACATCCGCTTGCTACTTTCTACACTTCATTATATATTGTGGTCAAGTAGT	1200
up	AAGGAGACCAACATCCGCTTGCTACTTTCTACACTTCATTATATATTGTGGTCAAGTAGT	1195
B1	AAGGAGACCAACATCCGCTTGCTACTTTCTACACTTCATTATATATTGTGGTCAAGTAGT	1195
CGN19839	AAGGAGACCAACATCCGCTTGCTACTTTCTACACTTCATTATATATTGTGGTCAAGTAGT	1200
30	AAGGAGACCAACATCCGCTTGCTACTTTCTACACTTCATTATATATTGTGGTCAAGTAGT	1200
61	AAGGAGACCAACATCCGCTTGCTACTTTCTACACTTCATTATATATTGTGGTCAAGTAGT	1200
422	AAGGAGACCAACATCCGCTTGCTACTTTCTACACTTCATTATATATTGTGGTCAAGTAGT	1200
L02	AAGGAGACCAACATCCGCTTGCTACTTTCTACACTTCATTATATATTGTGGTCAAGTAGT	1200
L03	AAGGAGACCAACATCCGCTTGCTACTTTCTACACTTCATTATATATTGTGGTCAAGTAGT	1200
S1003	AAGGAGACCAACATCCGCTTGCTACTTTCTACACTTCATTATATATTGTGGTCAAGTAGT	1200
S05	AAGGAGACCAACATCCGCTTGCTACTTTCTACACTTCATTATATATTGTGGTCAAGTAGT	1200
S06	AAGGAGACCAACATCCGCTTGCTACTTTCTACACTTCATTATATATTGTGGTCAAGTAGT	1200
WX2	AAGGAGACCAACATCCGCTTGCTACTTTCTACACTTCATTATATATTGTGGTCAAGTAGT	1200
S52	AAGGAGACCAACATCCGCTTGCTACTTTCTACACTTCATTATATATTGTGGTCAAGTAGT	1200
S94	AAGGAGACCAACATCCGCTTGCTACTTTCTACACTTCATTATATATTGTGGTCAAGTAGT	1200
SB-2	AAGGAGACCAACATCCGCTTGCTACTTTCTACACTTCATTATATATTGTGGTCAAGTAGT	1200
SD4	AAGGAGACCAACATCCGCTTGCTACTTTCTACACTTCATTATATATTGTGGTCAAGTAGT	1200
WD1	AAGGAGACCAACATCCGCTTGCTACTTTCTACACTTCATTATATATTGTGGTCAAGTAGT	1200
WD2	AAGGAGACCAACATCCGCTTGCTACTTTCTACACTTCATTATATATTGTGGTCAAGTAGT	1200
WX1	AAGGAGACCAACATCCGCTTGCTACTTTCTACACTTCATTATATATTGTGGTCAAGTAGT	1200
WZ1	AAGGAGACCAACATCCGCTTGCTACTTTCTACACTTCATTATATATTGTGGTCAAGTAGT	1200
	**	

```
WT          GGGTGGTCTCCAATATCGTTGACAAACCTGATTGGAGGCACACAAGTGAAGAAGGCATAT   1260
9930        GGGTGGTCTCCAATATCGTTGACAAACCTGATTGGAGGCACACAAGTGAAGAAGGCATAT   1260
up          GGGTGGTCTCCAATATCGTTGACAAACCTGATTGGAGGCACACAAGTGAAGAAGGCATAT   1255
B1          GGGTGGTCTCCAATATCGTTGACAAACCTGATTGGAGGCACACAAGTGAAGAAGGCATAT   1255
CGN19839    GGGTGGTCTCCAATATCGTTGACAAACCTGATTGC----ACACAAGTGAAGAAGGCATAT   1256
30          GGGTGGTCTCCAATATCGTTGACAAACCTGATTGGAGGCACACAAGTGAAGAAGGCATAT   1260
61          GGGTGGTCTCCAATATCGTTGACAAACCTGATTGGAGGCACACAAGTGAAGAAGGCATAT   1260
422         GGGTGGTCTCCAATATCGTTGACAAACCTGATTGGAGGCACACAAGTGAAGAAGGCATAT   1260
L02         GGGTGGTCTCCAATATCGTTGACAAACCTGATTGGAGGCACACAAGTGAAGAAGGCATAT   1260
L03         GGGTGGTCTCCAATATCGTTGACAAACCTGATTGGAGGCACACAAGTGAAGAAGGCATAT   1260
S1003       GGGTGGTCTCCAATATCGTTGACAAACCTGATTGGAGGCACACAAGTGAAGAAGGCATAT   1260
S05         GGGTGGTCTCCAATATCGTTGACAAACCTGATTGGAGGCACACAAGTGAAGAAGGCATAT   1260
S06         GGGTGGTCTCCAATATCGTTGACAAACCTGATTGGAGGCACACAAGTGAAGAAGGCATAT   1260
WX2         GGGTGGTCTCCAATATCGTTGACAAACCTGATTGGAGGCACACAAGTGAAGAAGGCATAT   1260
S52         GGGTGGTCTCCAATATCGTTGACAAACCTGATTGGAGGCACACAAGTGAAGAAGGCATAT   1260
S94         GGGTGGTCTCCAATATCGTTGACAAACCTGATTGGAGGCACACAAGTGAAGAAGGCATAT   1260
SB-2        GGGTGGTCTCCAATATCGTTGACAAACCTGATTGGAGGCACACAAGTGAAGAAGGCATAT   1260
SD4         GGGTGGTCTCCAATATCGTTGACAAACCTGATTGGAGGCACACAAGTGAAGAAGGCATAT   1260
WD1         GGGTGGTCTCCAATATCGTTGACAAACCTGATTGGAGGCACACAAGTGAAGAAGGCATAT   1260
WD2         GGGTGGTCTCCAATATCGTTGACAAACCTGATTGGAGGCACACAAGTGAAGAAGGCATAT   1260
WX1         GGGTGGTCTCCAATATCGTTGACAAACCTGATTGGAGGCACACAAGTGAAGAAGGCATAT   1260
WZ1         GGGTGGTCTCCAATATCGTTGACAAACCTGATTGGAGGCACACAAGTGAAGAAGGCATAT   1260
            **********************************     *********************

WT          CAAAAAGCAAGATTATGTCTCCACCCAGATAAGCTGCAGCAAAGAGGAGCGACAACGCTG   1320
9930        CAAAAAGCAAGATTATGTCTCCACCCAGATAAGCTGCAGCAAAGAGGAGCGACAACGCTG   1320
up          CAAAAAGCAAGATTATGTCTCCACCCAGATAAGCTGCAGCAAAGAGGAGCGACAACGCTG   1315
B1          CAAAAAGCAAGATTATGTCTCCACCCAGATAAGCTGCAGCAAAGAGGAGCGACAACGCTG   1315
CGN19839    CAAAAAGCAAGATTATGTCTCCACCCAGATAAGCTGCAGCAAAGAGGAGCGACAACGCTG   1316
30          CAAAAAGCAAGATTATGTCTCCACCCAGATAAGCTGCAGCAAAGAGGAGCGACAACGCTG   1320
61          CAAAAAGCAAGATTATGTCTCCACCCAGATAAGCTGCAGCAAAGAGGAGCGACAACGCTG   1320
422         CAAAAAGCAAGATTATGTCTCCACCCAGATAAGCTGCAGCAAAGAGGAGCGACAACGCTG   1320
L02         CAAAAAGCAAGATTATGTCTCCACCCAGATAAGCTGCAGCAAAGAGGAGCGACAACGCTG   1320
L03         CAAAAAGCAAGATTATGTCTCCACCCAGATAAGCTGCAGCAAAGAGGAGCGACAACGCTG   1320
S1003       CAAAAAGCAAGATTATGTCTCCACCCAGATAAGCTGCAGCAAAGAGGAGCGACAACGCTG   1320
S05         CAAAAAGCAAGATTATGTCTCCACCCAGATAAGCTGCAGCAAAGAGGAGCGACAACGCTG   1320
S06         CAAAAAGCAAGATTATGTCTCCACCCAGATAAGCTGCAGCAAAGAGGAGCGACAACGCTG   1320
WX2         CAAAAAGCAAGATTATGTCTCCACCCAGATAAGCTGCAGCAAAGAGGAGCGACAACGCTG   1320
S52         CAAAAAGCAAGATTATGTCTCCACCCAGATAAGCTGCAGCAAAGAGGAGCGACAACGCTG   1320
S94         CAAAAAGCAAGATTATGTCTCCACCCAGATAAGCTGCAGCAAAGAGGAGCGACAACGCTG   1320
SB-2        CAAAAAGCAAGATTATGTCTCCACCCAGATAAGCTGCAGCAAAGAGGAGCGACAACGCTG   1320
SD4         CAAAAAGCAAGATTATGTCTCCACCCAGATAAGCTGCAGCAAAGAGGAGCGACAACGCTG   1320
WD1         CAAAAAGCAAGATTATGTCTCCACCCAGATAAGCTGCAGCAAAGAGGAGCGACAACGCTG   1320
WD2         CAAAAAGCAAGATTATGTCTCCACCCAGATAAGCTGCAGCAAAGAGGAGCGACAACGCTG   1320
WX1         CAAAAAGCAAGATTATGTCTCCACCCAGATAAGCTGCAGCAAAGAGGAGCGACAACGCTG   1320
WZ1         CAAAAAGCAAGATTATGTCTCCACCCAGATAAGCTGCAGCAAAGAGGAGCGACAACGCTG   1320
            ************************************************************
```

WT	CAGAAACATGTTGCGGATAAGGCTTTTACCATCCTTCAGGAAGCGTGGTCTGTATATATA	1380
9930	CAGAAACATGTTGCGGATAAGGCTTTTACCATCCTTCAGGAAGCGTGGTCTGTATATATA	1380
up	CAGAAACATGTTGCGGATAAGGCTTTTACCATCCTTCAGGAAGCGTGGTCTGTATATATA	1375
B1	CAGAAACATGTTGCGGATAAGGCTTTTACCATCCTTCAGGAAGCGTGGTCTGTATATATA	1375
CGN19839	CAGAAACATGTTGCGGATAAGGCTTTTACCATCCTTCAGGAAGCGTGGTCTGTATATATA	1376
30	CAGAAACATGTTGCGGATAAGGCTTTTACCATCCTTCAGGAAGCGTGGTCTGTATATATA	1380
61	CAGAAACATGTTGCGGATAAGGCTTTTACCATCCTTCAGGAAGCGTGGTCTGTATATATA	1380
422	CAGAAACATGTTGCGGATAAGGCTTTTACCATCCTTCAGGAAGCGTGGTCTGTATATATA	1380
L02	CAGAAACATGTTGCGGATAAGGCTTTTACCATCCTTCAGGAAGCGTGGTCTGTATATATA	1380
L03	CAGAAACATGTTGCGGATAAGGCTTTTACCATCCTTCAGGAAGCGTGGTCTGTATATATA	1380
S1003	CAGAAACATGTTGCGGATAAGGCTTTTACCATCCTTCAGGAAGCGTGGTCTGTATATATA	1380
S05	CAGAAACATGTTGCGGATAAGGCTTTTACCATCCTTCAGGAAGCGTGGTCTGTATATATA	1380
S06	CAGAAACATGTTGCGGATAAGGCTTTTACCATCCTTCAGGAAGCGTGGTCTGTATATATA	1380
WX2	CAGAAACATGTTGCGGATAAGGCTTTTACCATCCTTCAGGAAGCGTGGTCTGTATATATA	1380
S52	CAGAAACATGTTGCGGATAAGGCTTTTACCATCCTTCAGGAAGCGTGGTCTGTATATATA	1380
S94	CAGAAACATGTTGCGGATAAGGCTTTTACCATCCTTCAGGAAGCGTGGTCTGTATATATA	1380
SB-2	CAGAAACATGTTGCGGATAAGGCTTTTACCATCCTTCAGGAAGCGTGGTCTGTATATATA	1380
SD4	CAGAAACATGTTGCGGATAAGGCTTTTACCATCCTTCAGGAAGCGTGGTCTGTATATATA	1380
WD1	CAGAAACATGTTGCGGATAAGGCTTTTACCATCCTTCAGGAAGCGTGGTCTGTATATATA	1380
WD2	CAGAAACATGTTGCGGATAAGGCTTTTACCATCCTTCAGGAAGCGTGGTCTGTATATATA	1380
WX1	CAGAAACATGTTGCGGATAAGGCTTTTACCATCCTTCAGGAAGCGTGGTCTGTATATATA	1380
WZ1	CAGAAACATGTTGCGGATAAGGCTTTTACCATCCTTCAGGAAGCGTGGTCTGTATATATA	1380
	**	

WT	TCTCAAGATGCCTTCATCAACTAA	1404
9930	TCTCAAGATGCCTTCATCAACTAA	1404
up	TCTCAAGATGCCTTCATCAACTAA	1399
B1	TCTCAAGATGCCTTCATCAACTAA	1399
CGN19839	TCTCAAGATGCCTTCATCAACTAA	1400
30	TCTCAAGATGCCTTCATCAACTAA	1404
61	TCTCAAGATGCCTTCATCAACTAA	1404
422	TCTCAAGATGCCTTCATCAACTAA	1404
L02	TCTCAAGATGCCTTCATCAACTAA	1404
L03	TCTCAAGATGCCTTCATCAACTAA	1404
S1003	TCTCAAGATGCCTTCATCAACTAA	1404
S05	TCTCAAGATGCCTTCATCAACTAA	1404
S06	TCTCAAGATGCCTTCATCAACTAA	1404
WX2	TCTCAAGATGCCTTCATCAACTAA	1404
S52	TCTCAAGATGCCTTCATCAACTAA	1404
S94	TCTCAAGATGCCTTCATCAACTAA	1404
SB-2	TCTCAAGATGCCTTCATCAACTAA	1404
SD4	TCTCAAGATGCCTTCATCAACTAA	1404
WD1	TCTCAAGATGCCTTCATCAACTAA	1404
WD2	TCTCAAGATGCCTTCATCAACTAA	1404
WX1	TCTCAAGATGCCTTCATCAACTAA	1404
WZ1	TCTCAAGATGCCTTCATCAACTAA	1404
	************************	
