# Supplementary material for: Mapping and identification of CsUp, a gene encoding an Auxilin-like protein, as a putative candidate gene for the upward-pedicel mutation (up) in cucumber
Source: BMC Plant Biol. 2019 Apr 25;19:157. doi: 10.1186/s12870-019-1772-4 (PMC6485165; doi:10.1186/s12870-019-1772-4)
Supplement: Supplementary file 9 — Figure S8. Genomic DNA sequence alignment of Cs535790 from WT and up (PDF 67 kb) [file 12870_2019_1772_MOESM9_ESM.pdf]

```
WT      ATGTCTTCTTCATCTGCAACTGCTTTCCATTCTTCTTTCTGTTTCTCTTCTCATAATAAA   60
up      ATGTCTTCTTCATCTGCAACTGCTTTCCATTCTTCTTTCTGTTTCTCTTCTCATAATAAA   60
        ************************************************************

WT      CTTTCTTCTTCTCCCTCAAATTCCCATCAACCAAACACTTCCTTATCACCCAAACCCAAA   120
up      CTTTCTTCTTCTCCCTCAAATTCCCATCAACCAAACACTTCCTTATCACCCAAACCCAAA   120
        ************************************************************

WT      ACCCTTCTCCATAAACATCCTCTCTACACGCCACTTCATTCCACAGTCTCTTCTCAAACC   180
up      ACCCTTCTCCATAAACATCCTCTCTACACGCCACTTCATTCCACAGTCTCTTCTCAAACC   180
        ************************************************************

WT      AAAGAGAAAATTCTCTGTCTTGAAATCATGGGTGTTGATTCTGGCAAAGCTCTTTCTCAA   240
up      AAAGAGAAAATTCTCTGTCTTGAAATCATGGGTGTTGATTCTGGCAAAGCTCTTTCTCAA   240
        ************************************************************

WT      AACCCTTCTCTTCATTCTGTTACTCTTGAATCCATTCACTCTGTCATCTCCTTCCTCCAA   300
up      AACCCTTCTCTTCATTCTGTTACTCTTGAATCCATTCACTCTGTCATCTCCTTCCTCCAA   300
        ************************************************************

WT      TCTAAAGGCATTCACCAAAAGGACTTCGCCAAAATCTTTGGAATGTGTCCTAAAATCCTC   360
up      TCTAAAGGCATTCACCAAAAGGACTTCGCCAAAATCTTTGGAATGTGTCCTAAAATCCTC   360
        ************************************************************

WT      ACTTCAGATGTTAAAACTGATTTAGTACCAGTTTTCAACTTTCTCTCTGAAGATCTCAAA   420
up      ACTTCAGATGTTAAAACTGATTTAGTACCAGTTTTCAACTTTCTCTCTGAAGATCTCAAA   420
        ************************************************************

WT      ATCCCAGATCAGAACTTTAGAAAAGCTATCAATAAGTGCCCAAGATTGCTTGCTTCAAGT   480
up      ATCCCAGATCAGAACTTTAGAAAAGCTATCAATAAGTGCCCAAGATTGCTTGCTTCAAGT   480
        ************************************************************

WT      GCTGAAGATCAGTTGAAACCTGCTTTGTTTTATCTTCAAAGACTTGGGTTGAAGGATTTG   540
up      GCTGAAGATCAGTTGAAACCTGCTTTGTTTTATCTTCAAAGACTTGGGTTGAAGGATTTG   540
        ************************************************************

WT      GAGGCTTTGGCTTATCATGATTCTGTTTTGCTGGTTTCAAGTGTGGAGAAGACCTTGATT   600
up      GAGGCTTTGGCTTATCATGATTCTGTTTTGCTGGTTTCAAGTGTGGAGAAGACCTTGATT   600
        ************************************************************

WT      CCTAAACTCAAGTATTTGGAGAGTTTGGGATTCACAAGGAGTGAAATCGTTGGGATGGTA   660
up      CCTAAACTCAAGTATTTGGAGAGTTTGGGATTCACAAGGAGTGAAATCGTTGGGATGGTA   660
        ************************************************************
```

```
WT    TTAAGGTGTCCAGCTCTGCTTACTTTCAGTATTGAAAACAATTTCAAGCCAAAGTTTGAG  720
up    TTAAGGTGTCCAGCTCTGCTTACTTTCAGTATTGAAAACAATTTCAAGCCAAAGTTTGAG  720
      ************************************************************

WT    TATTTCTCTGTGGAGATGCATAAAAAACTTGAGGAGCTTAAGGATTTTCCTCAGTATTTT  780
up    TATTTCTCTGTGGAGATGCATAAAAAACTTGAGGAGCTTAAGGATTTTCCTCAGTATTTT  780
      ************************************************************

WT    GCCTTTAGCTTGGAGAAGAGGATAAAGCCGAGGTATGTGGAGACAGTGGAGAGTGGAAAA  840
up    GCCTTTAGCTTGGAGAAGAGGATAAAGCCGAGGTATGTGGAGACAGTGGAGAGTGGAAAA  840
      ************************************************************

WT    AAAGTGCCTTTGTCACTTATGCTTAAGACCACAGATGTTGAGTTTAGAGAGTTGTTAGCA  900
up    AAAGTGCCTTTGTCACTTATGCTTAAGACCACAGATGTTGAGTTTAGAGAGTTGTTAGCA  900
      ************************************************************

WT    GAAGGGGGTGGTTGA 915
up    GAAGGGGGTGGTTGA 915
      ***************
```
