# Supplementary material for: Mapping and identification of CsUp, a gene encoding an Auxilin-like protein, as a putative candidate gene for the upward-pedicel mutation (up) in cucumber
Source: BMC Plant Biol. 2019 Apr 25;19:157. doi: 10.1186/s12870-019-1772-4 (PMC6485165; doi:10.1186/s12870-019-1772-4)
Supplement: Supplementary file 10 — Figure S9. Genomic DNA sequence alignment of Cs535810 from WT and up (PDF 43 kb) [file 12870_2019_1772_MOESM10_ESM.pdf]

```
WT    ATGGTGAAGATCGAAAGACAAAGAAGAGTCAGAGACGGAGGAGATGGAGGAGATCTCAGA   60
up    ATGGTGAAGATCGAAAGACAAAGAAGAGTCAGAGACGGAGGAGATGGAGGAGATCTCAGA   60
      ************************************************************

WT    TTAGGGGAGACAGAGGGATTGAGATTTGAGATCGGCCTGCGGAGGAGACAGGTGGACAGG   120
up    TTAGGGGAGACAGAGGGATTGAGATTTGAGATCGGCCTGCGGAGGAGACAGGTGGACAGG   120
      ************************************************************

WT    TCTAGGGTTGGATCGAAGATGTTGGAGGAGATGGATGCACAGGTCTCGAGTTGGATCTAA   180
up    TCTAGGGTTGGATCGAAGATGTTGGAGGAGATGGATGCACAGGTCTCGAGTTGGATCTAA   180
      ************************************************************

WT    ATATCAGAGGAGATGGACGGAGTTGAGAAGGAGGAGATGCCAGAGATGCTGCGAAGAGCG   240
up    ATATCAGAGGAGATGGACGGAGTTGAGAAGGAGGAGATGCCAGAGATGCTGCGAAGAGCG   240
      ************************************************************

WT    AGGGAGAGTAAGAGAGGTGAATTGTCTCGTGAGAAGTGTTGCACGAGGGAGAGCCGTGAG   300
up    AGGGAGAGTAAGAGAGGTGAATTGTCTCGTGAGAAGTGTTGCACGAGGGAGAGCCGTGAG   300
      ************************************************************

WT    GGAGATAGAAATCTAA   316
up    GGAGATAGAAATCTAA   316
      ****************
```
