# Supplementary material for: Mapping and identification of CsUp, a gene encoding an Auxilin-like protein, as a putative candidate gene for the upward-pedicel mutation (up) in cucumber
Source: BMC Plant Biol. 2019 Apr 25;19:157. doi: 10.1186/s12870-019-1772-4 (PMC6485165; doi:10.1186/s12870-019-1772-4)
Supplement: Supplementary file 11 — Figure S10. Alignment of promoter sequence of CsUP from WT and up. (PDF 108 kb) [file 12870_2019_1772_MOESM11_ESM.pdf]

```
WT    GTTTATAGATCAATTTTCTCTTTGAGTTGGCACAAAGAACGAATAGATAAAACTTGAAAA   60
up    GTTTATAGATCAATTTTCTCTTTGAGTTGGCACAAAGAACGAATAGATAAAACTTGAAAA   60
      ************************************************************

WT    CAACCAAAATTTTGGATAGCAAATTACACTTAAATGATTGTTCTATATTTATACTAAAAT   120
up    CAACCAAAATTTTGGATAGCAAATTACACTTAAATGATTGTTCTATATTTATACTAAAAT   120
      ************************************************************

WT    TAGATCATTTATGGTTTTTTTTATGAAATTTTAAAAGTCAATTTCTTTTATAATAATAAT   180
up    TAGATCATTTATGGTTTTTTTTATGAAATTTTAAAAGTCAATTTCTTTTATAATAATAAT   180
      ************************************************************

WT    TATGTGTATTTATTTCTTACAAAATATTTAAAATAATTTATTCTCATGTTAATTAAAACA   240
up    TATGTGTATTTATTTCTTACAAAATATTTAAAATAATTTATTCTCATGTTAATTAAAACA   240
      ************************************************************

WT    ACTTATGAACTTATATTTTTGAAGTTATGAAATTTGGAAAGCGAAACATATTTTGGTTGA   300
up    ACTTATGAACTTATATTTTTGAAGTTATGAAATTTGGAAAGCGAAACATATTTTGGTTGA   300
      ************************************************************

WT    GTGTAATTTATTTTTCATGTTTATTACAAACAACTTTTTCTATAGAAATCATTTATTTTC   360
up    GTGTAATTTATTTTTCATGTTTATTACAAACAACTTTTTCTATAGAAATCATTTATTTTC   360
      ************************************************************

WT    TATGTTACAAAAAAAAGTATTCACTACTATTTGATAATCAAAAAATGTAACTAAAGCATA   420
up    TATGTTACAAAAAAAAGTATTCACTACTATTTGATAATCAAAAAATGTAACTAAAGCATA   420
      ************************************************************

WT    CTAAAATACGTGTTTATAAAATATTTTATTTAGTAGTAGTTTATCAATAGTAAATTTGTT   480
up    CTAAAATACGTGTTTATAAAATATTTTATTTAGTAGTAGTTTATCAATAGTAAATTTGTT   480
      ************************************************************

WT    TATATGTTAATATTGAGTGTTTATAATAAATAATTTAATAATCAAAGAATGAAACAAATA   540
up    TATATGTTAATATTGAGTGTTTATAATAAATAATTTAATAATCAAAGAATGAAACAAATA   540
      ************************************************************

WT    TGTGTAGATGACAAATTTATAAATTGAAAACATGTTTAGAATTTATCAAATAGAATTTTT   600
up    TGTGTAGATGACAAATTTATAAATTGAAAACATGTTTAGAATTTATCAAATAGAATTTTT   600
      ************************************************************

WT    AAATTTACAATTCATAGGACAATATTGTAAATTTAGATTTTGGTAAGGGGTAGAAGCGTC   660
up    AAATTTACAATTCATAGGACAATATTGTAAATTTAGATTTTGGTAAGGGGTAGAAGCGTC   660
      ************************************************************
```

```
WT    CATTGAAAACATTAATGTGTAGAACAATATTGTTAATGTTTTTAAGTTGGCAAGAGGCAG   720
up    CATTGAAAACATTAATGTGTAGAACAATATTGTTAATGTTTTTAAGTTGGCAAGAGGCAG   720
      ************************************************************

WT    AATCATCCAAAAAGTGATTATTTTCTTAAGCGACTTTTAATATAGCAAGGATAAAAGGAA   780
up    AATCATCCAAAAAGTGATTATTTTCTTAAGCGACTTTTAATATAGCAAGGATAAAAGGAA   780
      ************************************************************

WT    AATTTTCATTTATAACCAAATAAATCAAAATATTTATAAAATATAGTAAATTTTTATATT   840
up    AATTTTCATTTATAACCAAATAAATCAAAATATTTATAAAATATAGTAAATTTTTATATT   840
      ************************************************************

WT    TTATAGATGATAACAACGATAGAATTTGATATACTACATTTCTTAATATAAATGATAACA   900
up    TTATAGATGATAACAACGATAGAATTTGATATACTACATTTCTTAATATAAATGATAACA   900
      ************************************************************

WT    AATATAGTAAATGTTTAATATAAAAGAAGCAAAAAAAGGAGGAACTTGGAATCAAAGTAT   960
up    AATATAGTAAATGTTTAATATAAAAGAAGCAAAAAAAGGAGGAACTTGGAATCAAAGTAT   960
      ************************************************************

WT    ATTAAACAAAAAAATAAAAACGTTAAGAAATGTAGTATGTCATATTATCACTTTTTTTTT   1020
up    ATTAAACAAAAAAATAAAAACGTTAAGAAATGTAGTATGTCATATTATCACTTTTTTTTT   1020
      ************************************************************

WT    AAATAAAGTTATGTATCGAGTAGTGATATATAAATTTATAAATGTGCAATACTATTACAT   1080
up    AAATAAAGTTATGTATCGAGTAGTGATATATAAATTTATAAATGTGCAATACTATTACAT   1080
      ************************************************************

WT    TTTCATTGATTCCATCGTTTTCAAATCTCTATGCTTGAACTTCCTACCAAACATACTTAA   1140
up    TTTCATTGATTCCATCGTTTTCAAATCTCTATGCTTGAACTTCCTACCAAACATACTTAA   1140
      ************************************************************

WT    ACTTGCACTTTTATGTCGGTACTTTCATTTTCTTAATTTTATTATAATTTTTTTAAAAAA   1200
up    ACTTGCACTTTTATGTCGGTACTTTCATTTTCTTAATTTTATTATAATTTTTTTAAAAAA   1200
      ************************************************************

WT    TGTATTGTAATAAATACATGAGTGACGATTTGAACTAGGATTTCTTGTCTACGAGTACAT   1260
up    TGTATTGTAATAAATACATGAGTGACGATTTGAACTAGGATTTCTTGTCTACGAGTACAT   1260
      ************************************************************

WT    ACAGATACCAGTTGAATTAAACTCATATTAGTTTCTCAATATGAAATTCAACAAGTTTGA   1320
up    ACAGATACCAGTTGAATTAAACTCATATTAGTTTCTCAATATGAAATTCAACAAGTTTGA   1320
      ************************************************************
```

```
WT      AATTTTTAATTGCATAGTATAGTAGGTTTAGACTATGGGTCTAATAAATCGTTTACCTAT   1380
up      AATTTTTAATTGCATAGTATAGTAGGTTTAGACTATGGGTCTAATAAATCGTTTACCTAT   1380
        ************************************************************

WT      TCTAACATTTTGAAAGTTGGTGTAGTAAAAAATAAAGTAAAAATCATAATTGAGTAGAAG   1440
up      TCTAACATTTTGAAAGTTGGTGTAGTAAAAAATAAAGTAAAAATCATAATTGAGTAGAAG   1440
        ************************************************************

WT      AACAATAACCAAATATAATATATTTATGACACGAAAAATATACTTAACATATATATATAT   1500
up      AACAATAACCAAATATAATATATTTATGACACGAAAAATATACTTAACATATATATATAT   1500
        ************************************************************

WT      ATGGGTATATTTGAGAATGTGGGTTTAGTGGTGGTTTGAGAAAGAGATCTAAACCTATTT   1560
up      ATGGGTATATTTGAGAATGTGGGTTTAGTGGTGGTTTGAGAAAGAGATCTAAACCTATTT   1560
        ************************************************************

WT      ATTACACTTTGAATCTTTTGTCTCAGAAATTGAAAACAAAGTATATTAGAAACATCATTT   1620
up      ATTACACTTTGAATCTTTTGTCTCAGAAATTGAAAACAAAGTATATTAGAAACATCATTT   1620
        ************************************************************

WT      TTAATTTATAAATTATTTTTTAAAGATCAGAAAATAAAAATAAAAAATAAAAAATAAAAA   1680
up      TTAATTTATAAATTATTTTTTAAAGATCAGAAAATAAAAATAAAAAATAAAAAATAAAAA   1680
        ************************************************************

WT      TCTCTTATTTTATTAAAGAAGAAAAAGGTCACACCTTTCTTTTAACTTTCTGCTCCACAT   1740
up      TCTCTTATTTTATTAAAGAAGAAAAAGGTCACACCTTTCTTTTAACTTTCTGCTCCACAT   1740
        ************************************************************

WT      GAATAGTTGTTCCAACTTTTTTTAAAAATAAACAAATAAACAAATAAACAAATACTAATT   1800
up      GAATAGTTGTTCCAACTTTTTTTAAAAATAAACAAATAAACAAATAAACAAATACTAATT   1800
        ************************************************************

WT      TATTATTATTATTATTAAACAGTAAAAAGTAGTTAACAGCTTTTTCCAATAATCCCACTT   1860
up      TATTATTATTATTATTAAACAGTAAAAAGTAGTTAACAGCTTTTTCCAATAATCCCACTT   1860
        ************************************************************

WT      TTTTGTTGTATAAATATATGAACGGCAGAGCTTCCATTTGTTTTCATGGTGTTTTCTCAT   1920
up      TTTTGTTGTATAAATATATGAACGGCAGAGCTTCCATTTGTTTTCATGGTGTTTTCTCAT   1920
        ************************************************************

WT      TCTCTCTCACTCTCTCACTCTTAAATCGTAATATTTCCTTCATTCTTTTTCATTTTTTCC   1980
up      TCTCTCTCACTCTCTCACTCTTAAATCGTAATATTTCCTTCATTCTTTTTCATTTTTTCC   1980
        ************************************************************
```

WT	CTCTTTTGTCTCCCCCGAAA	2000
up	CTCTTTTGTCTCCCCCGAAA	2000
	********************	
