# Supplementary material for: Mapping and identification of CsUp, a gene encoding an Auxilin-like protein, as a putative candidate gene for the upward-pedicel mutation (up) in cucumber
Source: BMC Plant Biol. 2019 Apr 25;19:157. doi: 10.1186/s12870-019-1772-4 (PMC6485165; doi:10.1186/s12870-019-1772-4)
Supplement: Supplementary file 12 — Figure S11. Alignment of promoter sequence of Csa1G535790 from WT and up. (PDF 109 kb) [file 12870_2019_1772_MOESM12_ESM.pdf]

```
WT    TGATATCAAGTAGGGTACCTAGCTACAAGAGTACTATTGATAGATTCACCTTTGTAAATG  60
up    TGATATCAAGTAGGGTACCTAGCTACAAGAGTACTATTGATAGATTCACCTTTGTAAATG  60
      ************************************************************

WT    TGGAAGTGAGAGTCGCTTCCTATGGAATTCTGGACAGATTCCTAGAGCATTCACTAAACT  120
up    TGGAAGTGAGAGTCGCTTCCTATGGAATTCTGGACAGATTCCTAGAGCATTCACTAAACT  120
      ************************************************************

WT    GGGATATACATGCAAGGCCATAAAAAGCATGGGCTAGTTTAGAACTTCACTGAAATAATA  180
up    GGGATATACATGCAAGGCCATAAAAAGCATGGGCTAGTTTAGAACTTCACTGAAATAATA  180
      ************************************************************

WT    TTGTGTGTGCTAGCATTAGAAGTAAAGTTCAAAACTACGAATTACTTTACGATATTTTAA  240
up    TTGTGTGTGCTAGCATTAGAAGTAAAGTTCAAAACTACGAATTACTTTACGATATTTTAA  240
      ************************************************************

WT    ACTATTGTCACTACGTAAAGATTCAAGTCATCAGACACCTTTACTTACACATAATATCAT  300
up    ACTATTGTCACTACGTAAAGATTCAAGTCATCAGACACCTTTACTTACACATAATATCAT  300
      ************************************************************

WT    AGAAACAGACACTAGGAGTGTAAGAATATGAGTTGAACCCAAATAACACGGACTACCCAA  360
up    AGAAACAGACACTAGGAGTGTAAGAATATGAGTTGAACCCAAATAACACGGACTACCCAA  360
      ************************************************************

WT    CCCATATTATAAGTGTTGGGTTGGGTTAAGTTAAAATATGAGTTGAATTGGGTTAAAAAA  420
up    CCCATATTATAAGTGTTGGGTTGGGTTAAGTTAAAATATGAGTTGAATTGGGTTAAAAAA  420
      ************************************************************

WT    ATTGACTTTTTTGGATTGGAGTGTTGAAATATTCGGTTCAACCCAACCCAACCCAACTCA  480
up    ATTGACTTTTTTGGATTGGAGTGTTGAAATATTCGGTTCAACCCAACCCAACCCAACTCA  480
      ************************************************************

WT    AATTAATATAGTATAATATATATATATATATATATATATATATATAGAAAAAAAAATTAGGT  540
up    AATTAATATAGTATAATATATATATATATATATATATATATATATAGAAAAAAAAATTAGGT  540
      ************************************************************

WT    ATATAAACTTTGAATTTATTGTATGAGATTTTGAATTATGTATGTATGAGATTTTGAATT  600
up    ATATAAACTTTGAATTTATTGTATGAGATTTTGAATTATGTATGTATGAGATTTTGAATT  600
      ************************************************************

WT    ATGTATGTTTGAAATTTAAATGCAACAATTTTGAACTTTTATAACCATTAGAACTAGAAA  660
up    ATGTATGTTTGAAATTTAAATGCAACAATTTTGAACTTTTATAACCATTAGAACTAGAAA  660
      ************************************************************
```

```
WT    TGAAAAAAAAAAAAAAAAAAAAACTATACAACCAGACAATTCAACCCAGCCTAACCCATA    720
up    TGAAAAAAAAAAAAAAAAAAAAACTATACAACCAGACAATTCAACCCAGCCTAACCCATA    720
      ************************************************************

WT    TTTTACGGGTTGAGTTAAGTTGGATTAGAAACTAAATTCGGGTTTGGTTGAGAATGTCTC    780
up    TTTTACGGGTTGAGTTAAGTTGGATTAGAAACTAAATTCGGGTTTGGTTGAGAATGTCTC    780
      ************************************************************

WT    TCAACCTAACCCAATTTCACCTCTCACAGACAACTCGATAGAAATGTTTTAAAAAAACTT    840
up    TCAACCTAACCCAATTTCACCTCTCACAGACAACTCGATAGAAATGTTTTAAAAAAACTT    840
      ************************************************************

WT    TCAATTGGGGGATTGATAGTAAACAAATTTCAAGTGGGGGATTAATGGATGAAAATTCTA    900
up    TCAATTGGGGGATTGATAGTAAACAAATTTCAAGTGGGGGATTAATGGATGAAAATTCTA    900
      ************************************************************

WT    TAAAAGGAAATATAATATTATTTTATTCTTAAAAGCATTTCCACATTGATTAATTTTGAT    960
up    TAAAAGGAAATATAATATTATTTTATTCTTAAAAGCATTTCCACATTGATTAATTTTGAT    960
      ************************************************************

WT    TGTGGATGTCCTTTCTTATACTCATTGGTTGATTAATGGGTTTGGTCTCGTAGGGCACCT    1020
up    TGTGGATGTCCTTTCTTATACTCATTGGTTGATTAATGGGTTTGGTCTCGTAGGGCACCT    1020
      ************************************************************

WT    AAATTTTGGAAGGAGGGTGGAGATAGGAAAATGTAGGTATGTTTGTATCTCAATGCCTCG    1080
up    AAATTTTGGAAGGAGGGTGGAGATAGGAAAATGTAGGTATGTTTGTATCTCAATGCCTCG    1080
      ************************************************************

WT    AGGTAAAATAATATCGCTCTCACAAACTATTATATATATCGTTTTCCCTTCATTCCTATA    1140
up    AGGTAAAATAATATCGCTCTCACAAACTATTATATATATCGTTTTCCCTTCATTCCTATA    1140
      ************************************************************

WT    AAATATCAAAACATTACTTTAGTATTCTCACTGAAGGTTCTCGAGCAAAATTGTGTATGT    1200
up    AAATATCAAAACATTACTTTAGTATTCTCACTGAAGGTTCTCGAGCAAAATTGTGTATGT    1200
      ************************************************************

WT    CGTTGAGTGATTTTATTTTGCTAGTTAATTTCCATGCAAAGTCAACGAGTGGAGAGTAGA    1260
up    CGTTGAGTGATTTTATTTTGCTAGTTAATTTCCATGCAAAGTCAACGAGTGGAGAGTAGA    1260
      ************************************************************

WT    ACTACGAAACGTCTTAAAGATAGCGTGTTCTCCCATCTACCGAGGCTATTTTTTGTTTGC    1320
up    ACTACGAAACGTCTTAAAGATAGCGTGTTCTCCCATCTACCGAGGCTATTTTTTGTTTGC    1320
      ************************************************************
```

```
WT      ACAGTCGAGAGTAGAACTACGAAACGTCTTAAAGATAGCGTGTTCTGCGACTCAGCCATC   1380
up      ACAGTCGAGAGTAGAACTACGAAACGTCTTAAAGATAGCGTGTTCTGCGACTCAGCCATC   1380
        ************************************************************

WT      TACTCATATAGTTTATGTTTTGCAGTAGTTTGATTCAATATTTTTCCTGTTGCTTATTGA   1440
up      TACTCATATCGTTTATGTTTTGCAGTAGTTTGATTCAATATTTTTCCTGTTGCTTATTGA   1440
        ********* **************************************************

WT      TGTTGTTGTTGTTGTCGTTTATTATTGTTGCGGCTACTATTTCTAATTATTTACTGTTCG   1500
up      TGTTGTTGTTGTTGTCGTTTATTATTGTTGCGGCTACTATTTCTAATTATTTACTGTTCG   1500
        ************************************************************

WT      TCGCTACTGAAACACTAAGACCGACGTGAATAGAAGATTTGCGTCGAAACAATCTCAACA   1560
up      TCGCTACTGAAACACTAAGACCGACGTGAATAGAAGATTTGCGTCGAAACAATCTCAACA   1560
        ************************************************************

WT      ATCACAAAATTGAAGCGTTGGAGAATACGTCATCGGGATAAAAATCTCGTCCAAACAATC   1620
up      ATCACAAAATTGAAGCGTTGGAGAATACGTCATCGGGATAAAAATCTCGTCCAAACAATC   1620
        ************************************************************

WT      CCACTTAAAATAAAAATTCAGACCAAATGCACCTATTAACACTAACAACTCAAGACTAAT   1680
up      CCACTTAAAATAAAAATTCAGACCAAATGCACCTATTAACACTAACAACTCAAGACTAAT   1680
        ************************************************************

WT      AAAAGGGTCATTTTTCAAAAAGAAAAAAAGAAAAAAAGAAAAAAAGAAAAAACCAACCAC   1740
up      AAAAGGGTCATTTTTCAAAAAGAAAAAAAGAAAAAAAGAAAAAAAGAAAAAACCAACCAC   1740
        ************************************************************

WT      GTAAAATAAAAATAAAATAAAATATTGAAATTAGAATGGCCTGACCCGATGGGTATGTCA   1800
up      GTAAAATAAAAATAAAATAAAATATTGAAATTAGAATGGCCTGACCCGATGGGTATGTCA   1800
        ************************************************************

WT      ACTAACTACTGTAAGGATAAAAAGTAATGAAATCCACCAACGAAGCTTGAGGCTTTTCTC   1860
up      ACTAACTACTGTAAGGATAAAAAGTAATGAAATCCACCAACGAAGCTTGAGGCTTTTCTC   1860
        ************************************************************

WT      ATCTCACCCAAACAAAACAATCTTTGAAATCCAACCTTAGCCAATCATCTTCCACCAAAA   1920
up      ATCTCACCCAAACAAAACAATCTTTGAAATCCAACCTTAGCCAATCATCTTCCACCAAAA   1920
        ************************************************************

WT      TTCCCCATAAGCTTAAATCCCCATTAGATTTATGTTTCTTCTTCACAAACAACACTTCAC   1980
up      TTCCCCATAAGCTTAAATCCCCATTAGATTTATGTTTCTTCTTCACAAACAACACTTCAC   1980
        ************************************************************
```

WT	TTCATCTCTCTCCTTCCACC	2000
up	TTCATCTCTCTCCTTCCACC	2000
	********************	
