# Supplementary material for: Mapping and identification of CsUp, a gene encoding an Auxilin-like protein, as a putative candidate gene for the upward-pedicel mutation (up) in cucumber
Source: BMC Plant Biol. 2019 Apr 25;19:157. doi: 10.1186/s12870-019-1772-4 (PMC6485165; doi:10.1186/s12870-019-1772-4)
Supplement: Supplementary file 13 — Figure S12. Alignment of promoter sequence of Csa1G535810 from WT and up. (PDF 108 kb) [file 12870_2019_1772_MOESM13_ESM.pdf]

```
WT    TAATAGTGATTTAACGTTAATAATAATACTTATAGTATATAGCAAATCAAACAACACGTA  60
up    TAATAGTGATTTAACGTTAATAATAATACTTATAGTATATAGCAAATCAAACAACACGTA  60
      ************************************************************

WT    TATTATAGTCGAATCATAATGATTTTTTGAAAGGTGTATATATCTTTTTCTTGAAAGAAA  120
up    TATTATAGTCGAATCATAATGATTTTTTGAAAGGTGTATATATCTTTTTCTTGAAAGAAA  120
      ************************************************************

WT    AAGGCAGCGTAGTTTATATTTATGTTAAGGCCCACCGACATAAAACACAAAATACAAACT  180
up    AAGGCAGCGTAGTTTATATTTATGTTAAGGCCCACCGACATAAAACACAAAATACAAACT  180
      ************************************************************

WT    TAATTAATTAGATATTAGGTTATTAAATTTTTTTAATTTTTGAAATGGAGAAATAATTGC  240
up    TAATTAATTAGATATTAGGTTATTAAATTTTTTTAATTTTTGAAATGGAGAAATAATTGC  240
      ************************************************************

WT    ATCTGTTATTGTCTCCTAATCTTTGAATTAAAGTTATTAATTGTTTGTTTTGCTAAAGAT  300
up    ATCTGTTATTGTCTCCTAATCTTTGAATTAAAGTTATTAATTGTTTGTTTTGCTAAAGAT  300
      ************************************************************

WT    TACAACCAAAAGGACATCAGATTTTCTTTTGTCCTCTTTTTTAATTCAAAAGCACAGACA  360
up    TACAACCAAAAGGACATCAGATTTTCTTTTGTCCTCTTTTTTAATTCAAAAGCACAGACA  360
      ************************************************************

WT    ACATTATTCTTTATGGATCCAACCACAACCAACATCATATCTTCACCTTTTATCAAATAT  420
up    ACATTATTCTTTATGGATCCAACCACAACCAACATCATATCTTCACCTTTTATCAAATAT  420
      ************************************************************

WT    TTACAACTTTCCTTATTTTCTTAACACCCATCAAATTTTAACATTCCCTAACAAATTGAT  480
up    TTACAACTTTCCTTATTTTCTTAACACCCATCAAATTTTAACATTCCCTAACAAATTGAT  480
      ************************************************************

WT    GACTTATGTCATAATATAATACACTTTTTCTTATTAAATATATGTTCAAAGATTATATTC  540
up    GACTTATGTCATAATATAATACACTTTTTCTTATTAAATATATGTTCAAAGATTATATTC  540
      ************************************************************

WT    CATGAGAAATACACATTTCAAATGATGATTCATTTGTCTTTATTTTATCGTCATTTTTTC  600
up    CATGAGAAATACACATTTCAAATGATGATTCATTTGTCTTTATTTTATCGTCATTTTTTC  600
      ************************************************************

WT    TAAATATAGATTGAAATGTGAAAAATATGTATGTATAGGGTTTGGATTTAAATCTTTTTC  660
up    TAAATATAGATTGAAATGTGAAAAATATGTATGTATAGGGTTTGGATTTAAATCTTTTTC  660
      ************************************************************
```

```
WT    TAAAATTAAAGATAAGAAAGAAGAGAAGGGCATTTGGTGACAATGCACTTGTGGTTGTGC   720
up    TAAAATTAAAGATAAGAAAGAAGAGAAGGGCATTTGGTGACAATGCACTTGTGGTTGTGC   720
      ************************************************************

WT    CCCATCAAGTCAACAGATGGATTTTTCTTGGTCACTAAATTAAAGCATTCAGAGAAAGTT   780
up    CCCATCAAGTCAACAGATGGATTTTTCTTGGTCACTAAATTAAAGCATTCAGAGAAAGTT   780
      ************************************************************

WT    CCTAGTTAAATACCATGTGATTTTAATTCCATTTTGTTGACCAAACTACAAATCTCACAC   840
up    CCTAGTTAAATACCATGTGATTTTAATTCCATTTTGTTGACCAAACTACAAATCTCACAC   840
      ************************************************************

WT    TTCTTTCCAACAATTAACTTACAAAAATATTTACTATTTCGACTTTAAATCAACGCCTAA   900
up    TTCTTTCCAACAATTAACTTACAAAAATATTTACTATTTCGACTTTAAATCAACGCCTAA   900
      ************************************************************

WT    CCTATGCTCTAAAAAAATTGATCAACTTAAGCTATATATATGTATATATAAACGTTCAAAC   960
up    CCTATGCTCTAAAAAAATTGATCAACTTAAGCTATATATATGTATATATAAACGTTCAAAC   960
      ************************************************************

WT    TTTAGATCCATCATTCCCTCGAAATTTAAAATAGGGAGACATTTTTTTTAATATATAATT   1020
up    TTTAGATCCATCATTCCCTCGAAATTTAAAATAGGGAGACATTTTTTTTAATATATAATT   1020
      ************************************************************

WT    TATAAATTTGTTGGCCTAAGTTCAAAATATATCTCCTCGACCTTTTTTTTATTTTTCCTA   1080
up    TATAAATTTGTTGGCCTAAGTTCAAAATATATCTCCTCGACCTTTTTTTTATTTTTCCTA   1080
      ************************************************************

WT    AGAAAAGCAAAATCTTATCTCAAACAAAGAAAAAAGCATTATGTACGGGACGACATGTAC   1140
up    AGAAAAGCAAAATCTTATCTCAAACAAAGAAAAAAGCATTATGTACGGGACGACATGTAC   1140
      ************************************************************

WT    TAGTAGTACTACAAAATTTTTCCTTTGCTAAAATACAGGATGTCTTTTCCAATTATAAAT   1200
up    TAGTAGTACTACAAAATTTTTCCTTTGCTAAAATACAGGATGTCTTTTCCAATTATAAAT   1200
      ************************************************************

WT    TATAAATAACATTTTTGTTTCATAAAATAATAAATATTAATTTCTACCTTTTTTTTAACT   1260
up    TATAAATAACATTTTTGTTTCATAAAATAATAAATATTAATTTCTACCTTTTTTTTAACT   1260
      ************************************************************

WT    TCAATGATACTCTAAAATTCTTTTAATCAAAATGTTGCTTTTTTTAATATTTGAAAAATA   1320
up    TCAATGATACTCTAAAATTCTTTTAATCAAAATGTTGCTTTTTTTAATATTTGAAAAATA   1320
      ************************************************************
```

```
WT    AATATAAAAAATAGGCAACACTGGAACCACATATTTTTTTAATAATGAATCAACCAACAT  1380
up    AATATAAAAAATAGGCAACACTGGAACCACATATTTTTTTAATAATGAATCAACCAACAT  1380
      ************************************************************

WT    TACCATATACGAGCAATTGTGTATATAAAAGTGCCACATATTTTTTTTTTAAAAATATGA  1440
up    TACCATATACGAGCAATTGTGTATATAAAAGTGCCACATATTTTTTTTTTAAAAATATGA  1440
      ************************************************************

WT    TAGTAAGAAAACCACATATTTGTAGATTATTTATAGAACACAAATAATCACATTAAAAAA  1500
up    TAGTAAGAAAACCACATATTTGTAGATTATTTATAGAACACAAATAATCACATTAAAAAA  1500
      ************************************************************

WT    AAAAAAAGAAGGAGAAAAAGCAAATCATTCTATTGAGTGTTGTGTCTTTTACCAAAAGAG  1560
up    AAAAAAAGAAGGAGAAAAAGCAAATCATTCTATTGAGTGTTGTGTCTTTTACCAAAAGAG  1560
      ************************************************************

WT    ATTATACCCTTTCGTGAATAATTGTGACTTTCTTTTAATAATATTTTTTGTAATGTTTTT  1620
up    ATTATACCCTTTCGTGAATAATTGTGACTTTCTTTTAATAATATTTTTTGTAATGTTTTT  1620
      ************************************************************

WT    CACGTGTTTTAACTCCAATTTTTTTTAAAGCCTAACATACTATTGTTCTATAAAAAAAAT  1680
up    CACGTGTTTTAACTCCAATTTTTTTTAAAGCCTAACATACTATTGTTCTATAAAAAAAAT  1680
      ************************************************************

WT    ATATATATATATTTTGTAAATGGAAAACGAGTGAAAATATTTTTAAAAAATAACAAAATT  1740
up    ATATATATATATTTTGTAAATGGAAAACGAGTGAAAATATTTTTAAAAAATAACAAAATT  1740
      ************************************************************

WT    TGAAAAGTTTTTTATAAAATCTAAAAAATGGTTATATTTTATAATTTTTTTGGTTAACTT  1800
up    TGAAAAGTTTTTTATAAAATCTAAAAAATGGTTATATTTTATAATTTTTTTGGTTAACTT  1800
      ************************************************************

WT    TGTTAGGTGTGTAATCGAGTTGGGTACACTTTCTCAACCCAACCCATACTTTTAGGTTAT  1860
up    TGTTAGGTGTGTAATCGAGTTGGGTACACTTTCTCAACCCAACCCATACTTTTAGGTTAT  1860
      ************************************************************

WT    TTGGGTTGACAATTTGAATTTAGTTTCCAACCAAACCAGTAAAATATGGGTTGTGTTGTC  1920
up    TTGGGTTGACAATTTGAATTTAGTTTCCAACCAAACCAGTAAAATATGGGTTGTGTTGTC  1920
      ************************************************************

WT    TTGTATAGTTTTTTTCCCCTAGTTTTCAATGGCTATAAAAGTTCAAAATCTTAGGTATAA  1980
up    TTGTATAGTTTTTTTCCCCTAGTTTTCAATGGCTATAAAAGTTCAAAATCTTAGGTATAA  1980
      ************************************************************
```

WT	TGATCTGACAGATATGAGAT	2000
up	TGATCTGACAGATATGAGAT	2000
	********************	
